# GLP-1 Use, Downstream Medical Spending, and Acute-Care Burden Among Adults with BMI-Defined Obesity: An Overlap-Weighted MEPS Analysis

**DOI:** 10.3390/healthcare14152362

**Published:** 2026-08-03

**Authors:** Onur Çelebi, Dilek Gümüş, Öner Gümüş

**Affiliations:** 1 PhD Economics Program, Institute of Social Sciences, Ankara University, 06560 Ankara, Turkey; ocelebi@ankara.edu.tr; 2 Department of Medical Services and Techniques, Vocational School of Health Services, Bilecik Şeyh Edebali University, 11210 Bilecik, Turkey; 3 Department of Accounting and Tax, Osmaneli Vocational School, Bilecik Şeyh Edebali University, 11500 Bilecik, Turkey; oner.gumus@bilecik.edu.tr; 4 Department of Accounting and Tax, Tavşanlı Vocational School, Kütahya Dumlupınar University, 43300 Kutahya, Turkey

**Keywords:** GLP-1 receptor agonists, BMI-defined obesity, medical expenditures, acute-care burden, inpatient utilization, pharmacoeconomics, overlap weighting, MEPS

## Abstract

**Background and Objective:** Glucagon-like peptide-1 (GLP-1) receptor agonist therapy has intensified the policy debate over whether obesity pharmacotherapy increases short-term healthcare expenditures or reduces downstream medical costs (i.e., non-drug, acute-care expenditures). This study estimated same-year associations between GLP-1 use and medical spending among adults with observed obesity. **Methods:** Analyses used Medical Expenditure Panel Survey (MEPS) person-year files compiled from 2018 to 2022, with body mass index (BMI)-defined analyses restricted to 2019 and 2021. The primary analytic sample included 7144 person-years, of which 275 represented sustained GLP-1 users. Overlap-weighted Poisson pseudo-maximum likelihood models, utilization regressions, two-part decompositions, and a layered diagnostic framework were applied to assess spending patterns and same-year cost implications. Robustness was evaluated through sensitivity analyses across all-obesity, severe-obesity, and diabetes-excluded subsamples, as well as exploratory molecule-specific specifications. **Results:** Findings showed no evidence of same-year cost savings, but rather a distinct shift in spending composition. In fully adjusted primary models, GLP-1 use was associated with reduced non-drug medical spending (beta = −0.2494; marginal effect = −$2586 per person-year) and lower acute-care spending (beta = −0.5856; marginal effect = −$2019), while total medical spending was higher (beta = 0.3182; marginal effect = +$6483). Point E values of 1.89 (non-drug) and 2.99 (acute-care) indicated moderate-to-strong resilience to unmeasured confounding. Negative control models using dental spending and visits showed significant positive associations. Since this residual confounding operates in the direction opposite to the negative acute-care association, the headline compositional pattern is unlikely to be an artifact of upward health-seeking bias. Utilization models indicated fewer inpatient discharges (IRR = 0.686) and inpatient nights (IRR = 0.524). Two-part models further suggested that the reduction in acute-care spending was concentrated on the intensive margin among individuals with positive expenditures. **Conclusions:** The use of GLP-1 is not associated with overall short-term cost savings. This usage is related to a shift in the composition of observed medical spending during the same year. This change is leading to a decline in hospital admissions and acute care costs, in parallel with the rise in pharmaceutical spending. Non-drug and acute-care costs were lower among regular users. However, total spending was at a higher level, consistent with a trend in the budget records from that period in which drug costs were the dominant factor. These associations are consistent with a potential reallocation of healthcare resources from inpatient acute care toward pharmacy and ambulatory services, with corresponding planning implications for payers and possible workforce reallocation of nursing capacity from acute to outpatient settings, contingent on replication in post-2022 data.

## 1. Introduction

Obesity has become a health-system financing and a clinical issue. The epidemiologic burden, as well as the economic impact, remains substantial and persistent on the international level, with the economic effects operating at several levels simultaneously: chronic disease management, inpatient care, nursing workload, outpatient intensity, prescription spending, labor-market impairment, and long-horizon fiscal pressure [1,2]. The cost burden of obesity has already been well established in the literature in economics, but the size and structure of the burden are highly subject to measurement and to the extent to which endogeneity is addressed. The instrumental-variables evidence itself and subsequent synthesis work both indicate that the actual medical cost burden of obesity may be underestimated by conventional descriptive estimates [3,4]. This issue has significantly increased in urgency due to GLP-1 receptor agonists. Obesity pharmacotherapy is no longer an adjunct to lifestyle management. The treatment landscape now includes guideline-backed pharmacologic management [5], established liraglutide efficacy data [6], large semaglutide obesity trials demonstrating significant weight loss [7,8], dual-incretin evidence from tirzepatide [9], and cardiovascular outcomes data that have broadened the expected value proposition of semaglutide beyond weight reduction alone [10]. The fundamental question for clinicians and payers is not whether these therapies decrease weight, but whether they change downstream medical use to a degree that alters spending composition in economically significant ways. This difference is important since the same-year budget impact and long-horizon value are not synonymous. The questions posed in model-based pharmacoeconomic studies often include whether the high acquisition costs can be compensated by lifetime quality-adjusted survival benefits, lower cardiometabolic morbidity, or lower cardiovascular morbidity [11,12,13]. An emerging body of real-world and claims-based evidence is beginning to demonstrate reduced inpatient and outpatient burden in semaglutide-treated patients, although this is a young field of research, often product-specific and rarely based on a nationally representative sample of the United States (US) population [14,15]. Therefore, this study asks a direct and policy-relevant question: Is GLP-1 use among US adults with BMI-defined obesity associated with a compositional shift in same-year medical spending across pharmacy, non-drug, and acute-care service lines? The empirical goal is non-causal by design. Despite this, MEPS has three properties that make it especially well-suited to the present question. It is nationally representative of the US civilian non-institutionalized population. It separates prescription spending from non-drug medical spending. And it reports the utilization outcomes that would mediate any compositional shift between downstream acute-care spending and contemporaneous pharmacy spending. We should not simply read our study window as a limitation on early data. It also reflects a clinically focused adoption period of GLP-1 therapy, preceding the subsequent wide proliferation of obesity-specific demand that was seen with later regulatory expansions (e.g., Wegovy in 2021, tirzepatide in diabetes mellitus (DM) in 2022 and obesity in 2023) [16,17,18]. During the same timeframe, the use of GLP-1 in MEPS remained embedded in high-risk cardiometabolic care, which is why the treated group was more clinically relevant to the portion of the health system that faces the greatest downstream burden.

This paper contributes to the body of literature on obesity and health economics in four aspects. First, it puts non-drug medical spending at the center of analysis as opposed to the use of total spending. This difference is economically important in that it is not merely whether drug therapy is costly; rather, it is whether more spending on pharmacy is associated with reduced downstream service-line costs in hospitals, emergency care and other non-drug aspects of medical utilization. Second, the research relies on observed adult BMI to conceptualize obesity and hence favors construct validity over diagnosis-code convenience. Third, it uses an overlap-weighted benchmark design and overlays other diagnostics—false-discovery-rate correction, raw-versus-adjusted contrasts, coefficient-retention analysis, sensitivity-concordance summaries, and offset decomposition—to demonstrate the extent to which the headline finding is robust to specification changes. Fourth, it shows that the most plausible channel of utilization runs through lower inpatient intensity, which significantly enhances the interpretation of lower acute-care spending. The manuscript does not assert cost savings in the same year. Its contribution is more specific: in the present MEPS analysis, the use of GLP-1 is associated with a compositional shift in observed same-year medical spending, with lower non-drug and acute-care spending accompanied by higher contemporaneous total spending. These findings carry direct implications for three audiences. For payer budget planning, contemporaneous pharmacy outlays must be weighed against lower inpatient burden. For health system workforce planning, a shift away from acute-care intensity may motivate the reallocation of nursing capacity from inpatient to ambulatory settings. For clinical decision-making, prescribers and care teams managing high-risk cardiometabolic populations need a clearer view of how same-year spending composition shifts under GLP-1 exposure. This interpretive stance is consistent with clinical evidence that the metabolic and cardiometabolic benefits of GLP-1 receptor agonists are not durably self-sustaining. Weight regain follows rapidly after discontinuation, and long-term maintenance depends on the integration of pharmacotherapy with structured lifestyle measures rather than on medication alone [19]. The same-year compositional shift documented here should therefore not be read as a permanent or self-financing cost offset, since the clinical benefit that plausibly drives the downstream acute-care reduction is itself contingent on sustained therapy.

## 2. Literature Review

### 2.1. Obesity as a Medical-Expenditure Problem

Three complementary literatures frame the present analysis. The first treats obesity not only as a clinical condition but also as a medical-expenditure and fiscal problem. Recent global surveillance confirms that the epidemiologic center of gravity has shifted decisively toward obesity: in most countries, the combined burden of underweight and obesity is now increasingly driven by obesity rather than undernutrition [1]. In parallel, Organization for Economic Cooperation and Development (OECD) analyses estimate that almost one in four adults in OECD countries is obese, that overweight and obesity absorb about 8.4% of total health spending across OECD systems, and that the US may devote nearly 14% of its health budget to the consequences of excess weight [2].

The cost literature in the US further demonstrates that the identification strategy, not just descriptive averages, determines the magnitude of the obesity burden. Cawley and Meyerhoefer calculated that obesity increases annual adult medical costs by about $2741 in 2005 dollars using an instrumental-variables design with MEPS data. This estimate is significantly higher than traditional correlation-based estimates, demonstrating how endogeneity and measurement error can significantly bias the estimated economic burden downward [3]. Kim and Basu, in a systematic review, meta-analysis, and empirical re-estimation, reported attributable annual spending of $1901 per obese adult in 2014 dollars, corresponding to $149.4 billion nationally, while also showing that age composition and adjustment for obesity-related comorbidities are major drivers of variation across estimates [4].

### 2.2. GLP-1 Therapeutics and Expected Economic Channels

Practice guidelines recommend adding pharmacotherapy to lifestyle intervention in adults with obesity when lifestyle treatment alone falls short [5]. Liraglutide 3.0 mg caused a mean weight loss of 8.4 kg at 56 weeks in SCALE Obesity and Prediabetes, compared to 2.8 kg with a placebo [6]. Semaglutide 2.4 mg decreased body weight by 14.9% at 68 weeks compared to 2.4% with a placebo in STEP 1, with 86.4% of treated individuals losing at least 5% of their body weight and 50.5% losing at least 15% [7]. These effects were not only temporary in STEP 5; the mean weight change at 104 weeks was −15.2% with semaglutide compared to −2.6% with placebo, suggesting long-lasting treatment effects under prolonged exposure [8]. By producing mean weight reductions of 15.0%, 19.5%, and 20.9% across the 5 mg, 10 mg, and 15 mg arms at week 72, compared to −3.1% with placebo, tirzepatide further extended the upper bound [9].

These trials are significant for the current study because they shed light on the direction of likely economic channels rather than because trial efficacy can be mechanically interpreted as cost savings. The earliest medical offsets should be anticipated in downstream high-cost care rather than as immediate reductions in overall spending if adiposity, glycemic risk, inflammation, and cardiometabolic strain are being altered to this extent. SELECT further showed a 20% reduction in major cardiovascular events with semaglutide versus placebo in this population [10]. Although this finding does not prove same-year savings on its own, it does support the hypothesis that near-term spending differences may first appear in acute care and inpatient utilization.

### 2.3. Where the Present Study Adds Value

Economic evaluation is the subject of the third literature, and the current body of evidence for the question this manuscript poses is still lacking. Cost-effectiveness ratios, scenario analyses, and long-term budget projections predominate in the pharmacoeconomic literature on tirzepatide and semaglutide, which is still largely model-based [11,12,13]. Although that work is helpful for payer forecasting, it is inherently assumption-intensive: outcomes rely on treatment duration, medication cost, rebound following discontinuation, and the mapping from intermediate risk-factor change to future morbidity. The immediate increase in drug costs and the high sensitivity of economic attractiveness to pricing and persistence assumptions are acknowledged by even favorable long-horizon analyses [12,13]. That literature provides background information but insufficient evidence for a paper interested in observed same-year service-line spending.

Although the body of real-world evidence is expanding, it also highlights the continued need for nationally representative expenditure decomposition. According to a recent narrative review, there are still few real-world GLP-1 receptor agonist studies in obesity, observed weight loss in practice is typically smaller than in trials, and discontinuation rates are high (often 20% to 50% within the first year), partly because real-world dosing, adherence, insurance access, and indication mix differ from trial conditions [14]. Those features matter economically because they decouple trial efficacy from realized spending offsets. Early claims-based utilization studies are beginning to report lower medical costs among semaglutide-treated high-risk obesity populations: one recent US study found 27% lower all-cause medical costs, 59% lower inpatient costs, and an estimated annual reduction of $3870 relative to matched non-users [15]. Yet such evidence remains claims-based, multimorbidity-focused, and not nationally representative. It therefore cannot substitute for a MEPS-based decomposition of total, non-drug, acute-care, utilization, and out-of-pocket spending in an analytic population defined by observed BMI. That empirical niche is precisely where the present study contributes.

## 3. Data and Methods

### 3.1. Data Source and File Assembly

The analysis uses person-year analytic files assembled from public use Medical Expenditure Panel Survey Full-Year Consolidated Data Files and linked event, prescribed-medicine, and condition files. MEPS is specifically designed to support policy-relevant research on expenditures, utilization, insurance coverage, and access for the US civilian noninstitutionalized population [20]. The documented extraction workflow supplied with this project indicates 146,994 person-year observations in the person-level extracts and 1,195,940 prescription-event rows before analytic restriction.

### 3.2. BMI-Defined Samples and GLP-1 Exposure

Observed adult BMI, not just obesity diagnosis codes, defines the primary analytic population. Adult BMI (ADBMI42) is only observed in 2019 and 2021 within the data construction workflow, which limits the calendar window despite being a significant measurement-strengthening decision. This calendar restriction is an explicit analytic trade-off. In other words, it strengthens construct validity by anchoring obesity classification to observed BMI rather than to diagnosis codes, but it narrows external breadth by restricting the analysis to two calendar years and by predating the post-2022 diffusion of obesity-focused GLP-1 and GIP therapies. We treat this trade-off as a substantive limitation and return to it in Section 6.

There are 275 sustained GLP-1 users among the 7144 person-years in the resulting primary sample. Three companion samples—an all-obesity sample (*n* = 11,078), a severe-obesity sample (*n* = 5047), and a DM-excluded sample (*n* = 8577)—are kept for sensitivity analysis. Because very few sustained GLP-1 users remain after DM-linked treatment is excluded, the DM-excluded sample is descriptively informative but underpowered for treated comparisons.

A documented molecule-matching workflow is used to calculate GLP-1 exposure from the MEPS prescription medication file. The resulting person-year GLP-1 file includes 1228 GLP-1 person-years overall, of which 1082 satisfy the sustained-use definition. Therefore, rather than being a clear indication of obesity alone, treatment in the BMI-defined primary sample is best understood as observed GLP-1 exposure in a mixed obesity and cardiometabolic treatment environment. Both interpretation and limitations depend on this distinction. No a priori sample-size calculation was performed; the analytic sample was determined by the availability of observed adult BMI in MEPS and the prevalence of sustained GLP-1 use within those survey years.

### 3.3. Outcomes, Covariates, and Survey Structure

Throughout this manuscript, acute-care burden refers collectively to both the spending and utilization dimensions of inpatient and emergency services; where the distinction matters, we use acute-care spending for the monetary outcome and inpatient utilization for the count-based outcome.

The primary outcome is non-drug medical spending, which is calculated by subtracting prescription costs from total medical spending. The question of whether the downstream medical-service burden decreases even when pharmacy spending increases mechanically makes this result economically instructive. Acute-care spending (inpatient plus emergency), overall medical spending, and out-of-pocket spending are additional spending outcomes. Emergency room visits, inpatient discharges, inpatient nights, office visits, and outpatient visits are examples of utilization outcomes. Distributional diagnostics demonstrate why this combination of models is suitable: acute-care spending has a very large mass at zero, while non-drug and total spending are generally positive for the majority of observations.

Age, age-squared, sex, race/ethnicity, calendar year, region, insurance coverage, poverty category, education, marital status, employment, self-rated health, self-rated mental health, and a clinically significant set of chronic-condition indicators are all examples of covariate adjustment. The raw descriptive evidence shows extremely large pre-weight imbalance in DM and substantial imbalance in BMI, dyslipidemia, hypertension, depression, self-rated health, and region; these are precisely the dimensions on which a serious adjustment strategy must work hardest. MEPS expenditure variables are subject to the survey’s standard editing and imputation procedures; all analytic variables in the final sample had complete data after standard MEPS processing, and no additional imputation was performed.

### 3.4. Econometric Framework

#### 3.4.1. Benchmark Overlap-Weighted Expenditure Models

The benchmark design combines overlap weighting with generalized linear expenditure models. Propensity scores are estimated for the probability of sustained GLP-1 use, conditional on the observed covariate set and calendar year. Overlap weights are then assigned as 1-e(x) for treated observations and e(x) for untreated observations, targeting the overlap population and reducing sensitivity to extreme propensity scores [21,22]. In plain terms, overlap weighting does two things at once. It up-weights individuals whose observed characteristics make them plausible candidates for either receiving or not receiving GLP-1 therapy. And it down-weights those whose covariate profiles place them clearly in one group. The net effect is to concentrate the analysis on the comparable region of the covariate space rather than on observations with little treated-control overlap. In accordance with standard diagnostic procedures, balance is assessed using absolute standardized mean differences [23].

Poisson pseudo-maximum likelihood (PPML) with a log link is the benchmark estimator for spending outcomes. The name refers to the Poisson distribution. Nevertheless, PPML is used here as a regression technique for non-negative, right-skewed continuous spending data, not as a count model. This works because PPML estimates the conditional mean directly on the dollar scale. It also accommodates observations with zero spending, without requiring a logarithmic transformation of the outcome. This decision avoids the retransformation issues that frequently occur in log-linear OLS models under heteroskedasticity and is in line with nonnegative, right-skewed spending data [24,25]. Additionally, the modeling decision is in line with more general recommendations on healthcare cost econometrics, which highlight the significance of estimators defined on the level scale of expected spending as opposed to just transformed outcomes [26]. Weighted Poisson models are used to estimate utilization outcomes, which are then compared to negative-binomial robustness. Two-part models that separate the probability of any spending from intensity among positive spenders are used to further analyze zero-heavy spending outcomes.

To clarify why PPML is preferred over alternative estimators common in the health-spending literature, we briefly outline the choice set considered for the benchmark spending models. (i) OLS on untransformed dollars violates the constant-variance and normality assumptions under right-skewed spending data and does not accommodate the multiplicative structure of the GLP-1 contrast. (ii) OLS on log-transformed dollars is sensitive to retransformation bias under heteroskedasticity, which is the source of inconsistency identified by Santos Silva and Tenreyro [24] and Manning and Mullahy [25] for log-linear specifications applied to nonnegative skewed outcomes; the issue is particularly relevant in spending data because the conditional variance commonly scales with the conditional mean. (iii) Gamma GLMs with a log link share PPML’s level-scale interpretation but require truncation or a two-part structure to accommodate zero spending, and the gamma assumption imposes a stronger constraint on the conditional variance than PPML’s quasi-likelihood framework. (iv) Tobit-type latent normal models impose a parametric censoring structure that is not justified for spending data, where zeroes represent genuine non-utilization rather than censored values. (v) Two-part models separate the extensive and intensive margins but aggregate the two stages only through a post-estimation step rather than through a single estimating equation. Therefore, PPML is selected as the primary estimator because it (a) estimates the conditional mean directly on the dollar scale, (b) is robust to heteroskedasticity in the multiplicative error structure typical of spending data, (c) accommodates zeroes without an ad-hoc transformation, and (d) yields marginal effects that are directly comparable across spending categories. The two-part decomposition and the negative-binomial robustness checks are reported as complementary specifications rather than competitors to the PPML benchmark.

Instead of using precise Taylor-linearized survey estimation, the present workflow uses survey-approximate inference. By this, we mean the following. The analysis incorporates MEPS survey weights and clustering structure into the estimation. However, it does so by combining them with the overlap weights and applying cluster-robust standard errors. It does not invoke the full Taylor-series variance machinery implemented in dedicated survey software. In particular, normalized products of the survey weight and overlap weight with cluster-robust variance at the VARSTR × VARPSU level are used in the final estimation. This is a reasonable approximation for the existing workflow, but rather than being a perfect substitute for a complete survey package implementation, it should be seen as a practical design-based compromise. We acknowledge that this approach is not the gold standard for survey data analysis: exact Taylor-linearized estimation, as implemented in dedicated survey software, would yield design-consistent variance estimates that more fully reflect the MEPS stratified, clustered sampling design. The point estimates reported here are unlikely to be materially affected, but the precision of the variance estimates, and therefore the width of confidence intervals and exact *p*-values, should be interpreted as approximate. We discuss this as a methodological limitation in Section 6 and identify it as a priority for future replication.

(1) $$e_{it}=Pr\left(T_{it}=1|X_{it},{year}_{t}\right)$$

(2) $$w_{it}=1-e_{it}\;\mathrm{for}\;\mathrm{treated};\;w_{it}=e_{it}\;\mathrm{for}\;\mathrm{untreated}$$

(3) $$E(Y_{it}|T_{it},X_{it},{year}_{t})=exp(\alpha +\beta \cdot T_{it}+X_{it}^{\prime }\gamma +\delta \cdot 1\left[year=2021\right])$$

A priori sample size calculation is not the appropriate transparency device for this design, because MEPS sample sizes are fixed by the survey rather than chosen by the researcher. That is why we report a post hoc minimum detectable effect (MDE) calculation that anchors the inferential capacity of the design to the realized analytic configuration. The primary sample contains *n* = 7144 person-years (sustained GLP-1 users n_t = 275; controls n_c = 6869). Under overlap weighting, the effective sample size (ESS) is approximated by E ≈ 4 × *n*_t × *n*_c/(*n*_t + *n*_c) ≈ 1057 person-years, which follows from the harmonic-mean structure of overlap weights when the propensity-score distribution is reasonably contained within the overlap region [27,28]. This closed-form harmonic mean expression is an upper bound approximation. The realized Kish effective sample size, computed from the actual analytic weights as (Σw)^2^/Σ(w^2^), is lower at 721 person-years (), with the gap between the two reflecting the departure of the realized weight distribution from the idealized harmonic mean benchmark. We use the realized Kish ESS of 721 as the operative precision figure. Under the spending side overdispersion typical of medical cost outcomes, this implies realized 80% power minimum detectable effects that vary by outcome (): approximately 34.4% for non-drug spending, 73.7% for acute-care spending, and 21.4% for total spending. The four primary spending coefficients reported compare directly against these thresholds. The non-drug and acute-care coefficients are economically large. The acute-care coefficients exceed their 80% power threshold, the non-drug coefficient sits just below its threshold, and the out-of-pocket coefficient is the least well-powered, consistent with the design being underpowered to detect modest contemporaneous shifts in patient cost sharing at the present sample size rather than with a true null effect. This MDE characterization is intended as a transparency device, not as a substitute for the formal inference reported alongside the point estimates.

#### 3.4.2. Diagnostic and Robustness Extensions

The benchmark design is expanded in five ways by the updated econometric application. The families of primary spending and utilization outcomes are first subjected to the Benjamini–Hochberg false discovery rate adjustment [29]. Second, the degree to which confounding influences the naive associations is revealed by directly comparing raw treated-control spending contrasts with fully adjusted overlap-weighted marginal effects. Third, as a straightforward indicator of specification stability, coefficient retention from the treatment-only model to the fully adjusted model is calculated. Fourth, an offset decomposition compares the observed non-drug and acute-care offsets with the implied contemporaneous drug-related increment. Fifth, to assess whether the sign pattern endures across analytic variations, a concordance summary aggregates the alternative specifications, including any-fill treatment, more general obesity definitions, severe-obesity limitations, and exploratory molecule-specific models. In addition, we implemented five diagnostic extensions in order to address potential biases and clarify precision: multi-method standard error validation (PSU clustering, stratum clustering, HC1, and nonparametric pairs-cluster bootstrap) to confirm inference stability; E-value sensitivity analysis [30] in order to quantify the strength of unmeasured confounding required to nullify the primary associations; Kish effective sample size (ESS) calculation to measure the precision loss induced by weighting; post hoc power and minimum detectable effect size (MDES) calculations (at 80% power and 5% significance) to characterize the inferential capacity of the design; and negative control falsification models using dental expenditure (DVTEXP) and dental visit count (DVTOT) as placebo outcomes.

**Figure 1:**
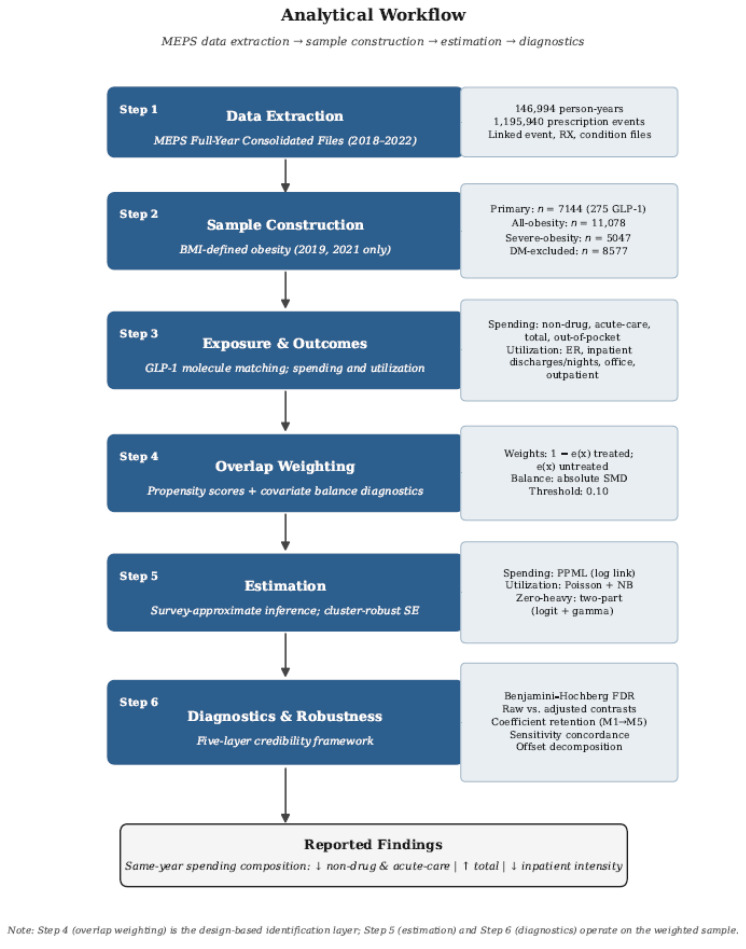
Analytical workflow from MEPS data extraction to estimation and diagnostics.

Figure 1 summarizes the full analytical workflow, from MEPS data extraction through sample construction, exposure and outcome definition, overlap weighting, estimation, and the diagnostics and robustness layer.

In order to complement Figure 1 with a concrete operational summary, the analytical pipeline can be expressed as the following high-level pseudocode; full step-by-step code, including package versions and helper functions, is provided in Appendix B.

## 4. Results

### 4.1. Sample Architecture and Raw Imbalance

**Figure A1:**
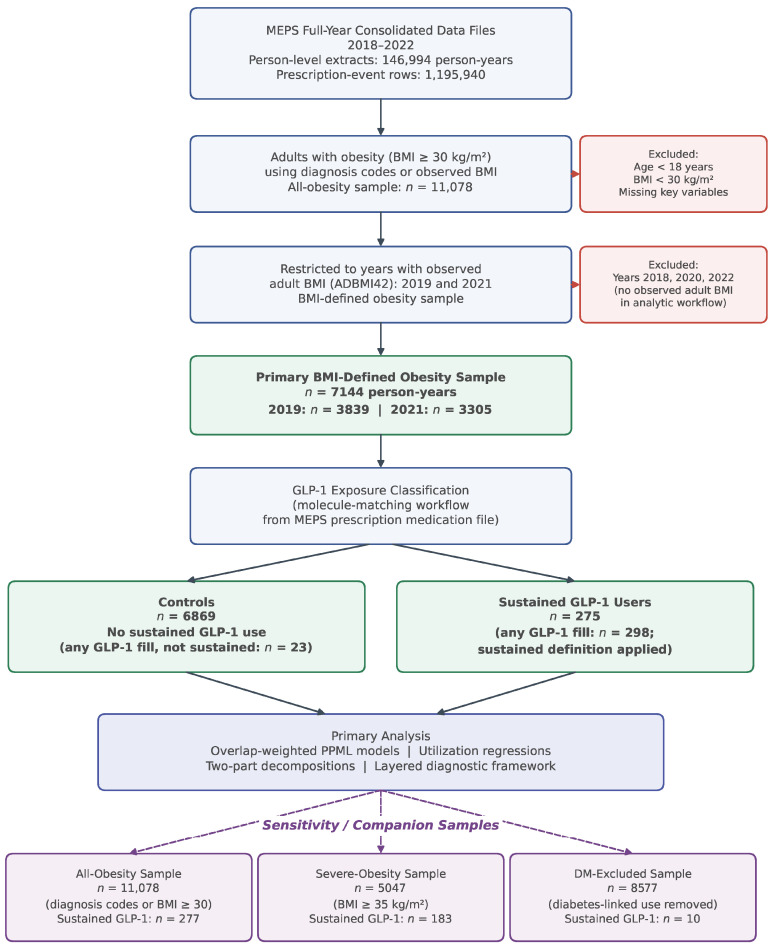
Participant flow diagram from the initial MEPS person-year extract to the primary BMI-defined analytic sample and sensitivity samples.

The sample architecture is shown in Table 1. The participant flow from the initial MEPS extract (146,994 person-years) to the final primary analytic sample (7144 person-years) is detailed in Figure A1. Every BMI-defined analytic sample is limited to the years 2019 and 2021 because adult BMI is only observed in those two years in the analytic workflow. A total of 7144 person-years are retained in the primary sample, comprising 6869 controls and 275 sustained GLP-1 users. The DM-excluded sample shows the practical identification limit of this dataset: treated observations become too sparse to support a strong main-design comparison once DM-linked use is screened out. In contrast, the all-obesity and severe-obesity samples offer larger or more clinically restrictive comparison sets.

Table 2 makes the raw selection problem explicit. Sustained GLP-1 users are older, materially heavier, more clinically burdened, and far more likely to have DM than controls. The DM standardized mean difference is 1.352 before weighting. Prescription spending is also sharply higher in treated observations, which is expected given the exposure definition, but raw total medical spending is correspondingly much higher as well. These descriptive contrasts are exactly why unadjusted spending comparisons cannot be interpreted as evidence about treatment-related offset.

### 4.2. Balance Improvement After Overlap Weighting

**Figure 2:**
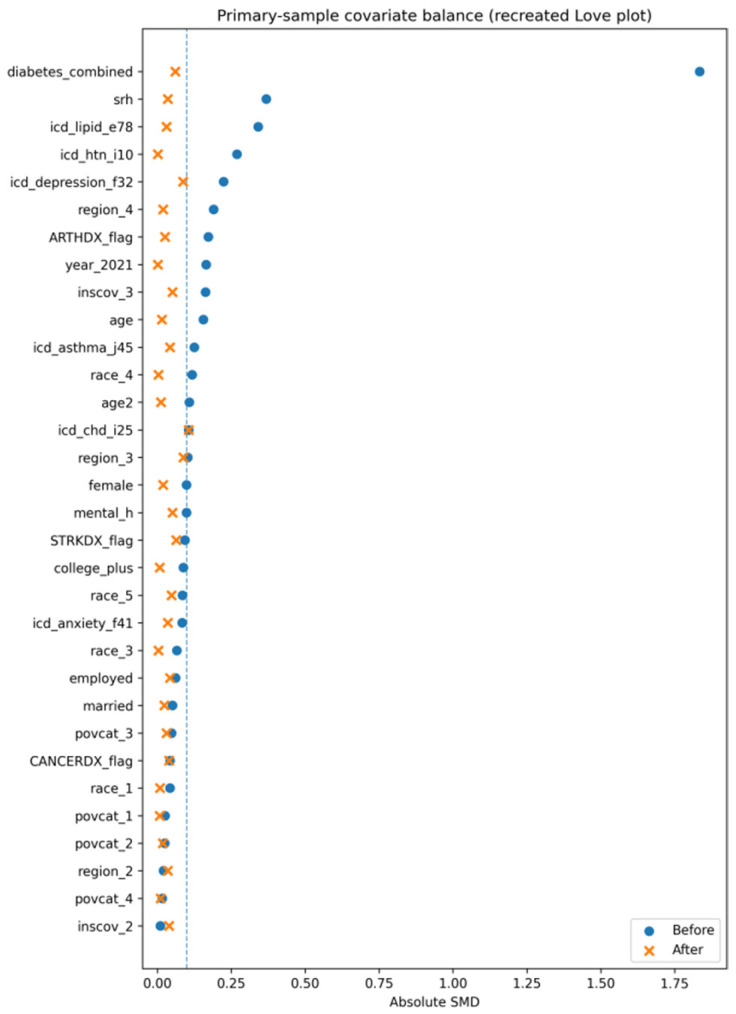
Primary-sample covariate balance before and after overlap weighting. Note: The dashed vertical line marks an absolute standardized mean difference of 0.10. Open circles denote raw imbalance, and × markers denote post-weight imbalance. The figure makes clear that DM remains the hardest covariate to balance, but the overall multivariate profile contracts sharply toward accepted balance thresholds.

Table 3 and Figure 2 show that overlap weighting materially improves observed balance, especially in the primary sample. The maximum absolute SMD falls from 1.8342 to 0.1058, and the mean post-weight SMD declines to 0.0333. The love plot indicates that DM remains the most challenging covariate, but the entire covariate profile contracts substantially toward the conventional 0.10 threshold. This is not proof of exchangeability; rather, it demonstrates that the overlap-weighted analysis is conducted in a markedly more comparable region of the covariate space than the raw treated-control comparison. DM in particular remains the most resistant covariate to balancing: its pre-weight SMD of 1.352 reflects the fact that, in this study window, GLP-1 use was strongly concentrated in patients with established cardiometabolic disease. Post-weight balance falls within conventional thresholds. However, the magnitude of the pre-weight imbalance means that residual confounding by indication, particularly through DM-linked baseline risk, cannot be fully ruled out by statistical adjustment alone. This concern is discussed further in Section 6.

### 4.3. Primary Spending Results

The central spending results appear in Table 4. In the fully adjusted primary model, GLP-1 use is associated with a 22.1% lower level of non-drug medical spending and a 44.3% lower level of acute-care spending, corresponding to marginal effects of −$2586 and −$2019 per person-year, respectively. At the same time, total medical spending remains 37.5% higher, or +$6483 per person-year. Out-of-pocket spending is directionally positive but not statistically significant after conventional testing or false-discovery-rate adjustment. These results imply a clear economic pattern: contemporaneous pharmacy costs are large enough to keep total spending above control levels, yet downstream non-drug and acute-care spending move in the opposite direction. Standard error robustness checks (Table 5) showed that the standard error estimates across the four methods were highly stable, differing by less than 10% for all primary outcomes. The nonparametric bootstrap standard errors (B = 500) were 15–20% smaller than the cluster robust standard errors, indicating that the bootstrap procedure yields somewhat smaller variance estimates under this specification. E-value calculations (Table 6) yielded point E-values of 1.89 (non-drug spending) and 2.99 (acute-care spending), with the E-values for the confidence interval limits closest to the null being 1.26 and 1.74, respectively. E-values were calculated from the PPML incidence rate ratios rather than from the dollar-scale average marginal effects. An unmeasured confounder would need to be associated with both treatment and outcome by risk ratios of at least 1.89 and 2.99, respectively, to explain away the observed point associations, and by at least 1.26 and 1.74 to explain away the confidence intervals. The realized effective sample size and the corresponding minimum detectable effects are reported in Table 7.

### 4.4. Credibility Diagnostics

**Figure 3:**
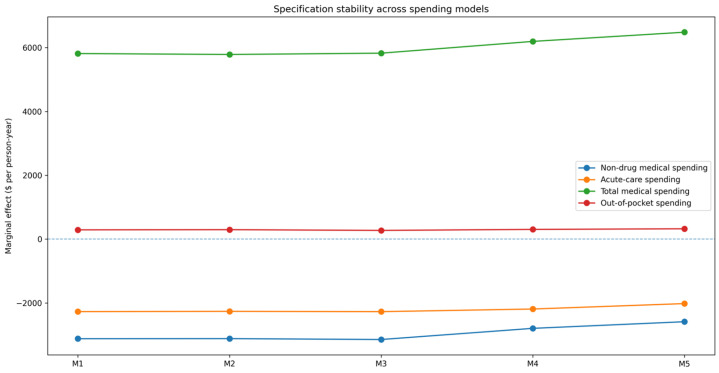
Specification stability across benchmark spending models. Note: M1 denotes the treatment-only model; M2–M5 sequentially add demographic, socioeconomic, and clinical controls. The key diagnostic is sign persistence: non-drug and acute-care effects remain negative throughout, total spending remains positive throughout, and out-of-pocket spending remains imprecisely positive. The horizontal dashed line marks zero, and colors distinguish the four spending outcomes as indicated in the legend.

Table 8 reveals how strongly confounding matters for interpretation. Raw non-drug medical spending is actually $1501 higher among GLP-1 users, but the fully adjusted marginal effect turns negative at −$2586, yielding the only sign reversal among the four major spending outcomes. The sign reversal is substantively important because it means naive descriptive comparisons would miss the main economic insight of the study. As Table 8 confirms, over 80% of the non-drug and acute-care effect magnitudes survive full covariate adjustment, while total spending grows modestly with added controls—consistent with positive selection on baseline medical need. This retention pattern across the M1-M5 specifications is displayed in Figure 3. 

As a further specification check, and in order to assess whether the headline pattern could be an artifact of generic differences in health-seeking behavior, we estimated identical overlap-weighted models for two negative-control outcomes that have no established mechanistic pathway to GLP-1 pharmacotherapy (Table 9). Both outcomes showed statistically significant positive associations with sustained GLP-1 use (dental spending β = 0.4069, *p* = 0.0161; dental visits β = 0.3994, *p* < 0.0001). These positive associations indicate that sustained GLP-1 users in 2019–2021 differed from non-users along unmeasured dimensions (most plausibly general health-seeking propensity and insurance generosity) that overlap weighting on the observed covariates does not fully remove. We therefore do not claim, on the basis of design alone, that the primary estimates are free of residual confounding. Critically, nonetheless, this residual confounding operates in the direction opposite to the headline acute-care finding. The placebo associations are positive, whereas the primary acute-care association is negative. Because the unmeasured factors that elevate dental utilization (broader health-seeking behavior, more generous coverage) would, if anything, also elevate general medical utilization, they would bias the acute-care contrast toward zero or toward positive values. The observation of a negative acute-care association despite this upward-biasing confounding structure suggests that the true cardiometabolic offset is at least as large as, and plausibly larger than, the benchmark estimate.

**Figure 4:**
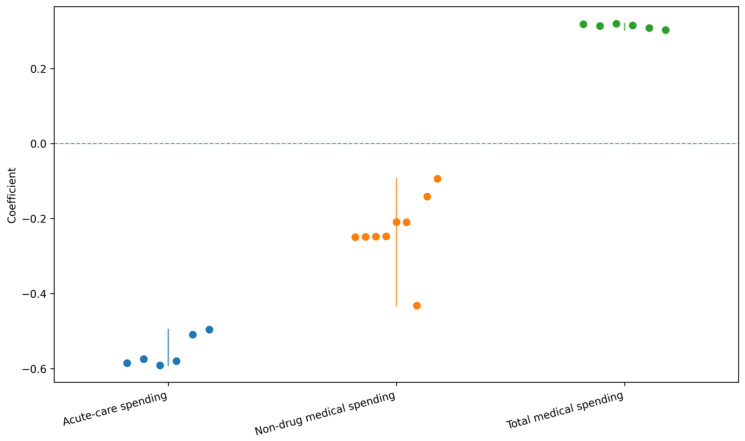
Sensitivity envelope across alternative specifications. Note: Filled circles denote individual alternative specifications, colored by outcome family as labeled on the horizontal axis; the vertical segment for each outcome spans the observed coefficient range across specifications, and the horizontal dashed line marks zero. Acute-care and total spending effects retain both sign and significance across specifications; non-drug medical spending retains its sign in every specification, with precision weakening in the more restrictive and molecule-specific analyses.

Figure 4 and the sensitivity concordance summary reinforce the same conclusion. Acute-care spending is negative across the alternative specifications examined and significant at both the 5% and 10% levels in each. Total medical spending is positive and significant across these specifications. Non-drug medical spending is uniformly negative, although precision weakens in the more restrictive severe obesity and molecule-specific subanalyses as treated cell sizes become smaller. We emphasize that these alternative specifications share the same underlying sample, exposure construction, and design, and differ only in sample restriction, exposure definition, or molecule subset. They should therefore be read as internal consistency checks on the sign pattern rather than as independent replications. With that caveat, the combination of stable sign and variable precision is the pattern one would hope to see in a small treated observational design.

### 4.5. Utilization Channels

The count models in Table 10 sharpen the mechanism. The strongest utilization signal is found on the inpatient margin: inpatient discharges have an IRR of 0.686, and inpatient nights have an IRR of 0.524 in the primary Poisson models, with corresponding negative-binomial estimates of 0.677 and 0.457. Emergency room visits are directionally lower but imprecisely estimated. Office visits are modestly higher, and outpatient visits are modestly lower, but neither pattern is as strong or as policy-relevant as the inpatient results. The most defensible mechanism reading is therefore not a generalized collapse in all service use. It is a specific attenuation of high-cost inpatient intensity.

This pattern is genuinely heterogeneous across utilization outcomes, and we report it as such. Of the five primary utilization contrasts, only inpatient discharges and inpatient nights achieve statistical significance after Benjamini–Hochberg adjustment (q = 0.0470 and q = 0.0100, respectively). The emergency room, office-visit, and outpatient-visit contrasts remain imprecise after FDR control (q ≥ 0.0954). Their point estimates do not move in a unified direction. Therefore, the mechanistic interpretation we develop in Section 5 should be read as inpatient-channel-specific (that is, as a description of the one utilization margin where the data speak clearly) rather than as a generalized statement about service use across all categories.

### 4.6. Two-Part Decomposition of Zero-Heavy Spending Outcomes

The two-part results in Table 11 deepen that interpretation. For acute-care spending, the probability of any positive spending is not statistically different, but conditional spending among positive spenders is markedly lower. The same pattern appears for inpatient and emergency components: no strong extensive-margin shift, but clearly lower intensity once spending occurs. This matters because it is consistent with a pattern in which the GLP-1 contrast does not operate at the extensive margin of acute-care utilization but is concentrated, instead, in the intensity of acute-care spending among individuals with positive expenditures. Out-of-pocket spending behaves differently: the probability of any out-of-pocket spending is higher, but the conditional amount among positive spenders is not precisely different, which is consistent with pharmacy exposure increasing the likelihood of some patient cost sharing without generating a large and robust increase in out-of-pocket intensity.

### 4.7. Sensitivity Concordance and Economic Interpretation

**Figure 5:**
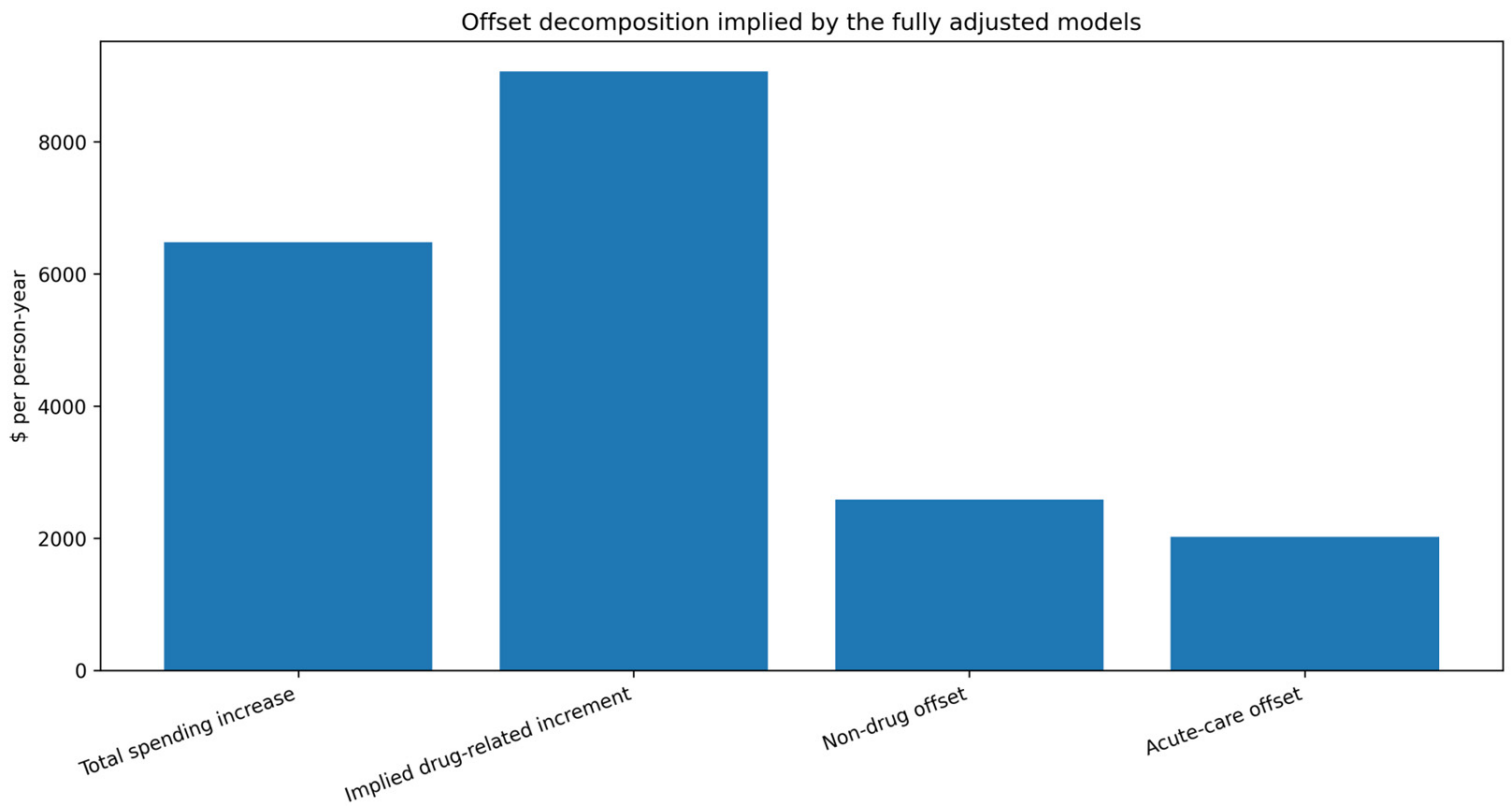
Illustrative offset decomposition implied by the fully adjusted models. Note: The implied drug-related increment is constructed as the fully adjusted total-spending increase plus the absolute non-drug offset. It should be interpreted as an accounting heuristic derived from separate marginal-effect models, not as a literal payer ledger.

**Figure 6:**
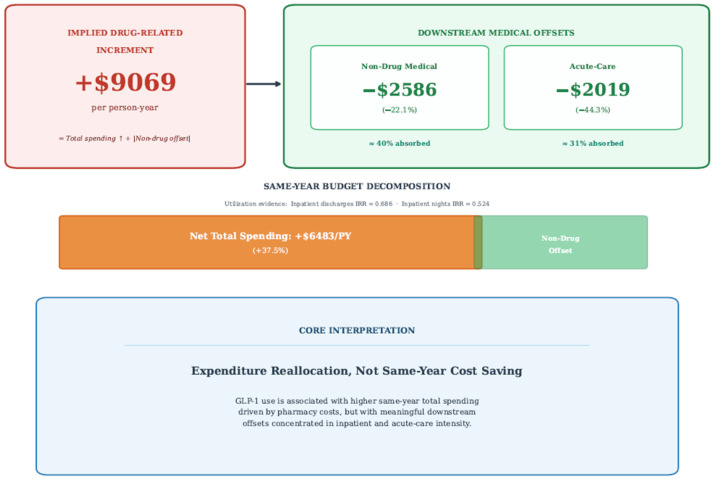
Same-year offset decomposition (Overlap-Weighted PPML Estimates • Primary BMI-Defined Sample • MEPS 2019–2021). Note: The implied drug-related increment is constructed as the fully adjusted total-spending increase plus the absolute non-drug offset. The downstream offsets show the non-drug medical (−$2586) and acute-care (−$2019) components. Net same-year total spending remains positive at +$6483 per person-year.

Figure 5 translates the primary spending results into a simple accounting interpretation. We note at the outset that this decomposition is illustrative rather than structural. In other words, the pharmacy, non-drug, and acute-care components are obtained from separate marginal effect models, each estimated on its own outcome with its own set of controls. Then, they are combined into a single visual ledger rather than estimated jointly within a unified accounting identity. That is why the shares displayed in Figure 5 should be read as a transparent summary of how the four primary marginal effects relate to one another in dollar terms, not as a structural decomposition derived from a single underlying budget equation. Subject to this caveat, the illustrative drug-related increment of $9069 per person-year is partially offset in the figure. This evidence means that the non-drug component absorbs roughly 40% and acute care absorbs 31%. Then, Figure 6 represents the same offset structure on the dollar scale, indicating that the lower non-drug (−$2586) and lower acute-care (−$2019) marginal effects partially offset the +$6483 same-year total spending. Nevertheless, the effects are not eliminated. Across Figure 5 and Figure 6, the pattern is consistent with a compositional shift in observed same-year medical spending rather than with a net same-year cost saving. Put differently, there is lower downstream non-drug and acute-care spending accompanied by higher pharmacy spending and higher total spending.

## 5. Discussion

In adults with BMI-defined obesity followed in MEPS, the use of GLP-1 correlates with reduced downstream non-drug medical spending and reduced acute-care spending despite an increase in contemporaneous total medical spending. Its main implication is conceptual more than empirical: in this context, same-year budget impact and downstream medical-burden reallocation do not denote the same thing, and the manuscript is most persuasive when it analytically distinguishes between the two objects. That interpretation fits the empirical evidence closely. Evidence from trials and guidelines demonstrates that the pharmacotherapy of obesity based on GLP-1 may significantly enhance the weight-related outcomes [5,6,7,8,9,10], whereas the pharmacoeconomic literature mostly converts those clinical results into long-horizon value statements or ratios between costs and effectiveness [11,12,13]. The current research is not in conflict with lifetime cost-effectiveness research. Rather, it adds nationally representative same-year data on the makeup of observed medical spending. To that extent, the paper is more aligned with budget-impact accounting and service-line redistribution than with complete welfare assessment, and that difference ought to be conspicuous in any journal framing. The most policy-relevant outcome of the manuscript is the inpatient channel. A reduction in acute-care spending is not only deduced by arithmetic subtraction across spending categories, but is also supported by utilization models that show a reduced number of inpatient discharges and a significantly smaller number of inpatient nights, as well as by two-part models that show that the difference in acute care is concentrated on the intensive margin among positive spenders. Since inpatient care represents one of the most policy-sensitive and expensive elements of downstream obesity burden, this overlap among outcome families significantly reinforces the meaning of the acute-care result. We label the inpatient-intensity channel as hypothesis-generating. The present design identifies an association between sustained GLP-1 use and the inpatient-spending margin. It does not isolate the clinical pathway through which that association arises. A direct test of mediation would require linked event-level data with diagnosis-coded admission reasons, longitudinal pre-treatment baselines, and a sample size adequate to support outcome decomposition by admission category, and none of these are available in the public use MEPS files used here. Hence, the inpatient-channel interpretation is the most parsimonious reading of the joint Section 4.4, Section 4.5 and Section 4.6 evidence. Nevertheless, it is offered as a candidate mechanism to be tested in linked-data extensions of this workflow rather than as a settled causal pathway. The supplementary diagnostics refine the trustworthiness instead of exaggerating certainty. The raw-versus-adjusted comparison illustrates the extent to which untreated descriptive contrasts would be misleading in this context, particularly in the case of non-drug spending where the naive treated-minus-control mean is positive, but the adjusted marginal effect is negative. Such reveals under methodologically appropriate estimation are a recurring phenomenon in applied economics. For instance, Gündem [31] shows that regional income-convergence findings that appear strong under classical panel estimators weaken sharply or disappear once spatial dependence is modeled.

The retention analysis demonstrates that the economically significant signs are robust across sequential covariate enrichment. The sensitivity envelope and concordance summary, a tabulation of how often the GLP-1 contrast retains the same sign and significance level across the alternative treatment, alternative sample, and molecule-specific specifications reported in Table 12, confirm that the core sign pattern persists across broader obesity definitions, severe-obesity restrictions, and exploratory molecule-specific specifications. None of these diagnostics eliminates the potential for residual confounding, but combined, they render the benchmark same-year design more transparent and much more plausible. The negative control falsification test reinforces this reading. The significant positive associations between sustained GLP-1 use and dental outcomes confirm directly, rather than merely conceding in principle, that residual confounding by unmeasured health-seeking propensity and insurance generosity is present in this sample. We report this transparently because a falsification test is informative only if its result is reported regardless of direction. At the same time, because the dental associations are positive while the acute-care association is negative, the residual confounding documented here works against the headline pattern rather than for it, which is why we interpret the benchmark acute-care offset as a lower bound on the cardiometabolic-specific effect rather than as an upper bound.

The positive same-year total spending relationship does not imply that the therapy lacks value; rather, it indicates that at the prices and diffusion schemes modeled in the present analysis, pharmacy costs dominate the contemporaneous budget ledger. This is apparent in the offset decomposition. The total-spending increment is fully adjusted with offsets that are sizable in non-drug and acute-care, although these offsets are not substantial enough to reverse the short-run pharmacy increment. The findings also carry direct implications for nursing practice and workforce planning. The observed shift away from inpatient intensity—fewer hospital discharges and shorter inpatient stays—implies a potential redistribution of nursing workload from acute inpatient settings toward ambulatory and outpatient care coordination. GLP-1 therapy requires structured patient education on injection technique, dose titration, gastrointestinal side-effect management, and long-term adherence support, all of which fall within the scope of nursing-led chronic disease management [32]. To the extent that GLP-1 exposure is associated with fewer high-acuity hospital episodes alongside greater demand for outpatient monitoring and self-management counseling, the compositional pattern documented in this study has a workforce corollary. Nursing resources may need to be reallocated, contingent on replication of the pattern in post-2022 data, from inpatient to ambulatory care models. This consideration is especially relevant for health systems that are simultaneously managing rising GLP-1 prescribing volumes and persistent inpatient nursing shortages. The findings also contribute to the discussion of external validity, and we want to be explicit about the boundaries of generalization that the analytic window imposes. Specifically, the 2019–2021 analytic window reflects an early-adoption, predominantly high-risk cardiometabolic prescribing environment rather than the broader obesity-treatment settings that have emerged after the post-2022 expansion of obesity-specific GLP-1 indications, the entry of tirzepatide, and the subsequent intensification of pricing competition; this distinction motivates the three extrapolation cautions developed below. The MEPS observation period analyzed here (2019 and 2021) reflects a pre-diffusion adoption regime: tirzepatide had not yet reached the market in either calendar year, semaglutide for chronic weight management (Wegovy) had only just received expanded US regulatory approval in mid-2021 and was not yet broadly diffused, and obesity-specific (rather than diabetes-led) GLP-1 prescribing was still a minority of total GLP-1 utilization. The composition pattern reported here therefore characterizes a clinically concentrated, cardiometabolic-driven user base operating under earlier list prices, earlier coverage rules, and earlier prescribing norms, and the magnitude documented in Table 4 and Table 6 should be read as benchmark anchors for that regime, not as point predictions for the contemporary market. It is a pre-tirzepatide limited-Wegovy-diffusion setting that is followed by means of a BMI-valid MEPS window. That is more conservative in analysis than some more recent real-world product-specific studies, but also less susceptible to selection bias on early adopters. In other words, the current work is informative on how exposure to GLP-1 was related to spending composition in a countrywide representative sample of obesity in a previous diffusion regime; it should not be overinterpreted as a definitive prediction of the contemporary market, nor dismissed as a simple descriptive anecdote. Three forms of extrapolation in particular should be avoided: (i) projecting the same-year offset magnitudes onto a post-2022 obesity-led prescribing population without re-estimation, since the marginal patient under broader access is likely to carry less baseline cardiometabolic risk and therefore smaller absolute downstream offsets in dollar terms; (ii) treating the pharmacy increment as fixed across pricing regimes, since net-of-rebate pharmacy outlays are a moving target subject to manufacturer pricing actions, payer rebate negotiations, and competitive entry; and (iii) generalizing molecule-specific patterns from the small treated cells observed here (semaglutide-only *n* = 62; liraglutide-only *n* = 118; dulaglutide-only *n* = 121) to current single-molecule prescribing volumes. The robust, transferable element of the present analysis is the directional pattern, same-year pharmacy increment partially offset by non-drug and acute-care reductions through an inpatient-intensity channel, rather than the specific dollar magnitudes, which should be re-anchored in any post-2022 replication. This generalization boundary is reinforced by the clinical trajectory of the field. The post-2022 prescribing environment is increasingly characterized by hybrid care models that pair GLP-1 pharmacotherapy with structured lifestyle and exercise components in order to address the cost, adherence, and long-term sustainability limitations of medication alone [19]. The same-year offset pattern observed in the present pre-diffusion, cardiometabolically concentrated window should accordingly be re-anchored, rather than assumed, in these later hybrid-management populations. From a nursing-research perspective, the same pre-diffusion window may also serve as a useful baseline against which future studies can measure how expanding GLP-1 access reshapes ambulatory nursing demand and inpatient staffing requirements. The paper also explains what this workflow can and cannot support methodologically. Overlap weighting, a propensity-score reweighting strategy that concentrates the analysis on individuals whose covariate profiles make them comparable candidates for either receiving or not receiving GLP-1 therapy, while down-weighting observations with little treated-control overlap (see Section 3.4.1), significantly enhances observed balance. Benjamini–Hochberg correction decreases the probability of false positives in related outcomes, and the sensitivity layer records sign stability. However, the design is observational and same year. Accordingly, the language used throughout the discussion section (including phrases such as “is associated with”, “co-occurs with,” and “is consistent with,” as well as any residual instances of “redistributes” or “reduces”) should be read uniformly as descriptions of an observational compositional pattern in a clinically concentrated, pre-diffusion adoption window, rather than as identification of a counterfactual treatment effect. Overlap weighting, sensitivity analysis, and the layered diagnostic framework strengthen the comparability of the benchmark contrast, but none of these procedures identifies a causal price elasticity or a treatment effect free of residual confounding. The results should be interpreted as a transparent benchmark association study, that is, a study whose primary task is to characterize how GLP-1 use tracks the composition of contemporaneous medical spending under a transparent comparability framework, rather than to identify a causal price elasticity or to estimate the long-run cost-offset trajectory, with exceptionally clear diagnostics, as opposed to hard causal evidence.

These findings suggest three sets of policy and operational considerations. We frame them as planning hypotheses derived from a pre-diffusion observational analysis, requiring confirmation in post-2022 data. First, for payers and pharmacy benefit managers, the documented composition pattern is consistent with reframing GLP-1 spending as a question of budget composition and with a contemporaneous budget framework that pairs pharmacy outlays with measured downstream offsets. Same-year budget impact analyses for GLP-1 coverage decisions should therefore

- project the pharmacy increment on the spending side and the non-drug and acute-care offsets on the savings side using the magnitudes documented here as benchmark anchors,
- explicitly distinguish same-year budget impact from long-horizon cost-effectiveness when communicating coverage decisions to plan sponsors and members, and
- integrate inpatient-utilization indicators, particularly inpatient discharges and inpatient nights, into formulary review committees as routine downstream metrics rather than treating spending in isolation.

Second, for health systems and hospital administrators, the inpatient-channel result has potential staffing model relevance. If sustained GLP-1 prescribing expands and the same-year pattern documented here is replicated in post-2022 data, planning frameworks may benefit from considering a reduction in cardiometabolic-driven inpatient demand and a corresponding increase in ambulatory follow-up, structured patient education on injection technique and gastrointestinal side effect management, and chronic disease nursing capacity. Operational steps that could be evaluated in this light include whether a share of any projected inpatient-day reduction might be redirected into ambulatory GLP-1 management, whether nursing-led titration and adherence models could be expanded, and whether inpatient nursing-demand forecasts should be updated to reflect the documented decline in inpatient discharges and nights among sustained users.

Third, for clinical decision-makers and prescribers, the same-year composition result supports framing GLP-1 therapy in high-risk cardiometabolic patients as associated with a contemporaneous compositional shift in observed medical spending rather than as an immediate cost-saving intervention. Clinical guidelines and shared decision making materials may accordingly describe the expected near-term spending profile (that is, higher pharmacy outlays accompanied by lower inpatient and acute-care intensity) as part of treatment-initiation conversations.

Across all three audiences, the central observation is the same: short-run payer budgets and downstream medical-burden management may move in opposite directions during the same calendar year, and policy and operational planning may benefit from being framed around that simultaneous, rather than sequential, pattern, subject to confirmation in post-2022 data.

## 6. Limitations

Several limitations remain important. First, the analysis is observational and repeated cross-sectional. Overlap weighting improves comparability on observed covariates, but unobserved confounding and confounding by indication remain plausible. The pre-weight DM imbalance is especially large, and treatment during this period often occurs in a cardiometabolic rather than obesity-only context. The pre-weight imbalance in DM is particularly severe (SMD = 1.352), reflecting the strong clinical concentration of GLP-1 use in patients with established diabetes during the study window. Although overlap weighting reduces this imbalance to within accepted thresholds, the magnitude of the baseline difference means that residual confounding, through both diagnosed DM and unmeasured cardiometabolic severity, may not be fully addressed by statistical adjustment. The likely direction of any such residual bias works against, rather than for, the headline pattern: GLP-1 users retain a higher unmeasured baseline medical need even after weighting, which would tend to inflate observed total spending and understate the observed reductions in non-drug and acute-care spending. The DM-excluded sensitivity sample partially addresses this concern by removing DM-linked treatment, but it is underpowered for inferential comparison. The headline same-year pattern should therefore be interpreted as a robust association in the overlap-weighted sample rather than as a causally identified treatment effect.

Second, the design is same-year. Exposure and outcomes are measured within the same person-year, so temporal ordering is imperfect. A serious next-stage design should exploit the official MEPS longitudinal panel structure and, where feasible, MEPS- National Health Interview Survey (NHIS) linkage resources to bring pre-treatment utilization, lagged spending, and richer baseline health measures into the adjustment set. Those resources were documented in the project materials, but merge-ready longitudinal microdata were not supplied in the present workflow.

Third, the BMI-defined analytic strategy trades external breadth for construct validity. Restricting to years with observed adult BMI strengthens obesity measurement relative to diagnosis-code-only approaches, but it narrows the study window to 2019 and 2021 and therefore predates the full diffusion of later agents and later reimbursement environments. As a result, the findings reported here characterize the same-year spending composition associated with GLP-1 use in a clinically concentrated, pre-diffusion adoption window; they should not be extrapolated mechanically to the broader, lifestyle-oriented prescribing environment that emerged after the 2021 expansion of semaglutide for chronic weight management and after the regulatory broadening of tirzepatide. The directional implication of this trade-off for the headline pattern is, however, conservative. This means that pre-diffusion GLP-1 users carried a heavier cardiometabolic burden and therefore a higher baseline acute-care risk than the average post-diffusion user. Hence, the observed inpatient and acute-care of offsets are best interpreted as evidence from a higher-risk population rather than as upper-bound estimates of the offset achievable in a broader lifestyle-oriented user base. Future replications using diagnosis code-defined samples or post-2022 BMI-augmented MEPS files should test whether the same composition pattern persists in later prescribing environments.

Fourth, subgroup power becomes limited outside the primary sample, especially in the DM-excluded and molecule-specific analyses. Finally, survey inference is approximate rather than exact Taylor-linearized design-based estimation, which should be improved in future replication. The current implementation combines normalized survey and overlap weights with cluster-robust variance at the VARSTR × VARPSU level, which captures the principal sources of design-based variability but does not reproduce the full Taylor-series variance decomposition implemented in dedicated survey software (e.g., Stata 19 or R 4.5.3). As a consequence, the precision of the variance estimates, and therefore the exact width of confidence intervals and the magnitude of *p*-values, should be interpreted as approximate, although the direction and order of magnitude of the point estimates are unlikely to be materially affected. A priority for future replication is to re-estimate the benchmark and sensitivity models using exact design-based survey estimation, ideally within an FSRDC-linked longitudinal extension of the present workflow.

Fifth, the offset decomposition reported in Section 4.7 and visualized in Figure 5 and Figure 6 is constructed from separate marginal-effect estimates. The four primary spending outcomes are each estimated in their own overlap-weighted PPML model, with their own covariate-adjustment trajectory and their own cluster-robust variance. Then, the resulting marginal effects are combined into a single visual ledger. This approach is transparent and easy to interpret. However, it does not satisfy the exact adding-up restriction emerging from a structural budget identity in which the pharmacy, non-drug, and acute-care components are estimated jointly. Since the components are jointly estimated, the offset percentages reported in Figure 5 are illustrative summaries of how four separately estimated marginal effects relate to one another in dollar terms. A priority for future replication is to re-estimate the offset structure within a unified framework that imposes cross-component restrictions. Both should be tested for the robustness of the illustrative shares reported here and permit formal inferences based on the offset coefficients themselves rather than the four marginal effects in isolation.

The restriction to 2019 and 2021 narrows calendar breadth, but it also strengthens internal interpretability. This period predates the later mass diffusion of obesity-focused GLP-1/glucose-dependent insulinotropic polypeptide (GIP) therapy and therefore captures a more clinically concentrated prescribing environment. In that sense, the present estimates are best interpreted as evidence from a baseline cardiometabolic adoption era, when GLP-1 users were more likely to represent high-risk patients with substantial metabolic burden rather than a broad lifestyle-oriented population.

We implemented the negative control falsification test using dental outcomes (DVTEXP and DVTOT) directly within the public use MEPS files instead of deferring it to future restricted-access work. The test turned statistically significant positive associations for both placebo outcomes, confirming the presence of residual confounding through health-seeking behavior and insurance generosity. Since this bias is positive and the headline acute-care association is negative, the documented confounding operates against, not in favor of, the primary finding; the placebo result therefore characterizes the residual confounding environment without overturning the headline pattern, and it supports interpreting the acute-care offset as conservative rather than inflated. A separate structural constraint concerns longitudinal identification. A within-person longitudinal design would, in principle, strengthen identification beyond the same-year cross-sectional contrast reported here. However, it cannot be constructed for this exposure-outcome configuration from the public use files since BMI is observed only in the 2019 and 2021 questionnaire waves and not as a continuous within-person panel series. A priority for future replication is therefore an FRSDC-linked, NHIS-MEPS longitudinal extension (e.g., NHMEP19X/NHMEP21X), which would permit both a within-person design and an expanded negative control layer with diagnosis-coded admission categories.

## 7. Conclusions

Among adults with BMI-defined obesity in MEPS, GLP-1 use is associated with less non-drug medical spending, less acute-care spending and lower inpatient intensity, despite a higher level of concomitant total medical spending. The interpretation most consistent with the evidence is not same-year cost saving but a same-year compositional shift in observed medical spending: higher pharmacy spending is accompanied by, rather than offset by, lower downstream non-drug and acute-care spending. That distinction matters for both scholarship and policy. This is because it is erroneous to reduce the economic evidence to the single question of whether GLP-1 therapy saves money; for policy, this is because short-run payer budgets and downstream medical burden management may move in opposite directions within the same year. A therapy may be associated with higher current-year costs while co-occurring with lower non-drug and inpatient spending in ways that are important both clinically and operationally. Accordingly, the main value that the paper has to offer is not to provide definitive causal savings, but rather a robust, policy-relevant benchmark pattern: higher same-year total spending accompanied by lower downstream non-drug and acute-care spending, with the most prominent contributing channel concentrated in the inpatient-intensity component. Future work should test that pattern in official MEPS longitudinal panels, and where possible in richer, pre-treatment linkage designs. Until then, the current evidence is consistent with an element of caution: the use of GLP-1 is associated with greater pharmacy outlays on the margin but is also consistent with a more favorable composition of downstream medical use. Given the observational design and the early, diabetes-concentrated adoption window analyzed here, the policy and healthcare planning implications of these findings should be regarded as provisional and confirmed in post-2022 data before informing coverage or planning decisions. For nursing, these results may motivate anticipation of workforce reallocation from acute inpatient care toward structured ambulatory GLP-1 management, patient education, and adherence support as prescribing volumes continue to expand.

## Figures and Tables

**Table 1:** Analytic sample architecture.

Analytic Sample	Total *n*	Sustained GLP-1	Controls	Any GLP-1 Fill	2019	2021
Primary BMI-defined obesity sample	7144	275	6869	298	3839	3305
All-obesity sensitivity sample	11,078	277	10,801	300	6232	4846
Severe-obesity sensitivity sample	5047	183	4864	196	2816	2231
DM-excluded sensitivity sample	8577	10	8567	11	4935	3642

Notes: BMI = body mass index (kg/m^2^); DM = diabetes mellitus; GLP-1 = glucagon-like peptide-1 receptor agonist. Sample sizes are reported as person-years from the MEPS Full-Year Consolidated files (2019, 2021). The primary sample restricts to adults with a BMI ≥ 30 kg/m^2^ and at least one cardiometabolic risk factor; all-obesity applies to a BMI ≥ 30 alone; severe obesity applies to a BMI ≥ 35; DM-excluded restricts to a BMI ≥ 30 in the absence of diagnosed diabetes. All BMI-defined samples are limited to 2019 and 2021 because observed adult BMI is only available in those years in the analytic workflow. Treated observations become sparse after DM-linked use is eliminated. For this reason, the DM-excluded sample is kept for transparency but is not used as the primary design. Estimates derived from this sample are presented descriptively in the sensitivity panel and should be interpreted as underpowered relative to the primary, all-obesity, and severe-obesity samples.

**Table 2:** Selected unweighted characteristics in the primary sample.

Characteristic	GLP-1 Users	Controls	SMD
Age, years	60.03	58	0.155
BMI, kg/m^2^	38.72	36.18	0.41
Non-drug medical spending, $	9769.71	8268.44	0.086
Total medical spending, $	25,189.32	11,674.41	0.514
Acute-care spending, $	2648.06	2816.75	0.017
Prescription spending, $	15,419.61	3405.98	0.687
Office visits, count	15.64	10.67	0.276
Female, %	61.1	56.2	0.098
DM, %	97.1	32.5	1.352
Hypertension, %	40	27.4	0.267
Dyslipidemia, %	34.2	19.3	0.337
Coronary heart disease, %	5.5	3.3	0.106
Depression, %	12.7	6.2	0.223
Self-rated health (1–5)	3.25	2.89	0.369

Notes: Continuous variables are reported as means. Binary variables are reported as percentages. SMD = standardized mean difference between treated and control groups, computed as the difference in means divided by the pooled within-group standard deviation; |SMD| ≤ 0.10 is the conventional acceptable-balance threshold. BMI = body mass index (kg/m^2^); DM = diabetes mellitus. Currency values are in US dollars per person-year. The table is diagnostic rather than inferential: its purpose is to show the intensity of pre-weight treated-control selection, especially for DM, prescription spending, BMI, and self-rated health.

**Table 3:** Balance diagnostics across weighted analytic samples.

Analytic Sample	Total *n*	Sustained GLP-1	Max SMD Before	Max SMD After	Mean SMD After
Primary BMI-defined obesity sample	7144	275	1.8342	0.1058	0.0333
All-obesity sensitivity sample	11,078	277	2.4001	0.1164	0.0355
Severe-obesity sensitivity sample	5047	183	2.0291	0.1615	0.038

Notes: Total *n* = total number of person-years in the analytic sample; Sustained GLP-1 = number of person-years classified as sustained GLP-1 users (≥2 fills or ≥60 days’ supply); max SMD before/max SMd after = maximum absolute standardized mean difference across the full covariate vector specified in Section 3, computed before and after applying overlap weights w = 1 − e(x) for treated and w = e(x) for controls, where e(x) is the estimated propensity score; Mean SMD after = mean absolute SMD across the same covariate factor after weighting. The conventional balance threshold is |SMD| ≤ 0.10; values are absolute (sign-free). GLP-1 = glucagon-like peptide-1 receptor agonist; BMI = body mass index (kg/m^2^). The primary sample achieves the strongest post-weight balance profile and is therefore the principal evidentiary design. Post-weight maximum SMDs should be interpreted together with the raw baseline imbalance; the point of overlap weighting here is to improve comparability in the overlap population, not to claim elimination of all residual confounding.

**Table 4:** Fully adjusted primary-sample spending results with Benjamini–Hochberg adjustment.

Outcome	β	Robust SE	% Change	Marginal Effect ($/PY)	*p*-Value	BH q-Value
Non-drug medical spending	−0.2494	0.1056	−22.1	−2586	0.0181	0.0241
Acute-care spending	−0.5856	0.1973	−44.3	−2019	0.003	0.006
Total medical spending	0.3182	0.0693	37.5	6483	0	0
Out-of-pocket spending	0.1766	0.1147	19.3	325	0.1237	0.1237

Notes: Estimates are from M5 (fully adjusted) overlap-weighted Poisson pseudo-maximum-likelihood (PPML) specifications using the survey-approximate variance implementation, with cluster-robust standard errors at the VARSTR × VARPSU level (primary sample, *n* = 7144 person-years). β = log-link coefficient on sustained GLP-1 use; Robust SE = cluster-robust standard error; % change = exp (β) − 1, expressed as a percentage; Marginal effect; ($/PY) = average treated-minus-control predicted spending contrast in US dollars per person-year; *p*-value = raw two-sided *p*-value; BH q-value = Benjamini–Hochberg-adjusted q-value [28], calculated across the family of our primary spending outcomes. PPML = Poisson pseudo-maximum likelihood; GLP-1 = glucagon-like peptide-1 receptor agonist.

**Table 5:** Standard error robustness validation across alternative variance estimators.

Outcome	Beta	SE Cluster (VARSTRxVARPSU)	SE Cluster (VARSTR)	SE HC1	SE Bootstrap (B = 500)
nondrug_medexp	−0.2494	0.1056 (*p* = 0.0181)	0.1020 (*p* = 0.0145)	0.1094 (*p* = 0.0227)	0.0867 (*p* = 0.0040)
acute_care_exp	−0.5856	0.1973 (*p* = 0.0030)	0.2053 (*p* = 0.0043)	0.2084 (*p* = 0.0050)	0.1571 (*p* = 0.0000)
total_medexp	+0.3182	0.0693 (*p* = 0.0000)	0.0680 (*p* = 0.0000)	0.0696 (*p* = 0.0000)	0.0562 (*p* = 0.0000)
oop_exp	+0.1766	0.1147 (*p* = 0.1237)	0.1090 (*p* = 0.1052)	0.1091 (*p* = 0.1055)	0.0906 (*p* = 0.0280)

Notes: All models are overlap-weighted PPML specifications using sample_A_primary (*n* = 7144, treated = 275). SE Cluster (VARSTR × VARPSU) represents the benchmark primary survey-approximate approach. SE Cluster (VARSTR) aggregates clusters at the stratum level. SE HC1 is heteroskedasticity-robust without survey design clustering. SE Bootstrap reports nonparametric paired cluster bootstrap standard errors (B = 500 replications resampling PSUs within strata).

**Table 6:** E-value sensitivity analysis for unmeasured confounding.

Outcome	Beta	Rate Ratio (RR)	CI Bound (Closest to Null)	E-Value (Point)	E-Value (CI Bound)
nondrug_medexp	−0.2494	0.7792	0.9584	1.89	1.26
acute_care_exp	−0.5856	0.5568	0.8196	2.99	1.74
total_medexp	+0.3182	1.3747	1.2001	2.09	1.69
oop_exp	+0.1766	1.1932	0.9530	1.67	1.28

Notes: E-values represent the minimum strength of association (expressed as a Risk Ratio) that an unmeasured confounder must have with both the treatment (GLP-1 use) and the expenditure outcome to explain away the observed beta coefficient, beyond the observed covariates. Conversion from PPML coefficients to Rate Ratios (RRs) uses exp(beta). E-values are calculated on the RR scale rather than from average marginal effects. Calculations follow VanderWeele & Ding [30].

**Table 7:** Overlap-weighted effective sample size (ESS) and post-hoc power (MDES).

Group	Nominal *n*	ESS (Overlap-Weighted)	ESS Ratio (%)	Outcome	Observed SE	MDES (α = 0.05, 80% Power)	Observed Beta	Post-Hoc POWER
Overall	7144	721	10.1%	nondrug_medexp	0.1056	≥0.2957 (34.4%)	−0.2494 (Significant)	65.6%
Treated (GLP-1)	275	207	75.2%	acute_care_exp	0.1973	≥0.5524 (73.7%)	−0.5856 (Exceeds MDES)	84.3%
Control (non-GLP-1)	6869	1260	18.3%	total_medexp	0.0693	≥0.1940 (21.4%)	+0.3182 (Exceeds MDES)	99.6%
-	-	-	-	oop_exp	0.1147	≥0.3212 (37.9%)	+0.1766 (Below MDES)	33.7%

Notes: Effective sample size (ESS) is calculated using Kish’s formula: ESS = (Σw)^2^ /Σ (w^2^). The ESS ratio is relative to nominal sample N. The minimum detectable effect size (MDES) is calculated at the 5% significance level (two-sided, alpha = 0.05) and 80% target power as 2.80 ∗ SE, with the percentage change calculated as exp(MDES)-1. The z-score critical values used are z_alpha/2 = 1.96 and z_beta = 0.84. ‘Significant’ indicates that the observed absolute beta coefficient exceeds the significance threshold (1.96 ∗ SE), and ‘Exceeds MDES’ indicates it exceeds the 80–power MDES threshold (2.80 ∗ SE). Observed standard errors are design-adjusted and cluster-robust (VARSTR × VARPSU level). Post hoc power is calculated as cdf(|beta|/SE − 1.96).

**Table 8:** Raw versus fully adjusted spending contrastsand coefficient retention from treatment-only to fully adjusted models.

Outcome	M1 Marginal ($/PY)	Absolute Effect Retained (%)	Raw Mean Difference ($/PY)	M5 (Fully Adjusted Marginal) ($/PY)	Sign Reversal
Non-drug medical spending	−3118	82.9	1501	−2586	Yes
Acute-care spending	−2270	88.9	−169	−2019	No
Total medical spending	5815	111.5	13,515	6483	No
Out-of-pocket spending	291	111.8	867	325	No

Notes: Raw mean difference (USD/PY) = treated-minus-control mean difference from the primary descriptive workbook, in US dollars per person-year; Fully adjusted marginal (USD/PY) = M5 (fully adjusted) overlap-weighted PPML marginal-dollar estimate from the benchmark primary models (also reported in Table 4). Sign reversal = “Yes” indicates that the naive descriptive contrast and the fully adjusted model imply opposite directions, signaling that the confounding structure is large enough to flip the substantive interpretation; “No” indicates that the adjusted estimate retains the same sign as the raw difference, even if the magnitude changes substantially. M1 marginal (USD/PY) = treatment-only marginal-dollar contrast for sustained GLP-1 use, in US dollars per person-year; M5 marginal (USD/PY) = corresponding fully adjusted marginal-dollar contrast (also reported in Table 4). Absolute effect retained (%) = |M5|/|M1| × 100; high retained shares imply that the economically relevant sign and a large share of magnitude survive the sequential addition of demographic, socioeconomic, and clinical controls. PPML = Poisson pseudo-maximum likelihood; GLP-1 = glucagon-like peptide-1 receptor agonist.

**Table 9:** Negative control falsification PPML model results.

Outcome	Variable	Beta	Robust SE	*p*-Value	Significance	95% CI	Marginal Effect	Mean(Y)	Zero %
Dental expenditure	DVTEXP	0.4069	0.1690	0.0161	**	[0.076, 0.738]	+$250	$410	59.6%
Dental visit count	DVTOT	0.3994	0.0982	0.0001	***	[0.207, 0.592]	+0.51 visits	1.0	59.1%

Notes: Results are derived from Poisson pseudo-maximum likelihood (PPML) models estimated using the identical covariate adjustment (M5) and overlap-weighting structure as the primary spending models. Standard errors are PSU-clustered and robust. Significance: *** *p* < 0.01, ** *p* < 0.05. Marginal effects represent average dollar or visit count differences per person-year.

**Table 10:** Primary-sample utilization models with FDR-adjusted significance.

Outcome	Poisson IRR	95% CI Low	95% CI High	*p*-Value	BH q-Value	NB IRR	NB *p*-Value
Emergency room visits	0.876	0.638	1.202	0.4107	0.4107	0.809	0.1593
Inpatient discharges	0.686	0.501	0.94	0.0188	0.047	0.677	0.0158
Inpatient nights	0.524	0.347	0.79	0.002	0.01	0.457	0
Office visits	1.135	0.987	1.307	0.0763	0.0954	1.134	0.0595
Outpatient visits	0.758	0.566	1.015	0.063	0.0954	0.718	0.0139

Notes: Estimates are from overlap-weighted Poisson regressions of person-year utilization counts on the sustained-GLP-1 indicator and the M5 covariate set, with cluster-robust standard errors at the VARSTR × VARPSU level (primary sample, *n* = 7144 person-years). Poisson IRR = incidence rate ratio computed as exp (β) on the GLP-1 indicator from the overlap-weighted Poisson regression. These are the benchmark estimates. 95% CI low/95% CI high = lower and upper bounds of the cluster-robust 95% confidence interval for the IRR; *p*-value = raw two-sided *p*-value; BH q-value = Benjamini–Hochberg-adjusted q-value [29], calculated across the family of five primary utilization outcomes; NB IRR/NB *p*-value = corresponding incidence rate ratio and *p*-value from the negative-binomial robustness specification, reported as a distributional robustness check. GLP-1 = glucagon-like peptide-1 receptor agonist.

**Table 11:** Two-part decomposition for zero-heavy spending outcomes.

Outcome	Part 1 Logit Coef	Part 1 OR	Part 1 *p*-Value	Part 2 Gamma Coef	Part 2% Change	Part 2 *p*-Value	Positive Spenders
Acute-care spending	−0.2103	0.8103	0.2242	−0.5404	−41.7	0.001	1760
Out-of-pocket spending	1.2775	3.5877	0.0093	0.1561	16.9	0.1031	6503
Inpatient spending	−0.1526	0.8585	0.4466	−0.5944	−44.8	0.0003	860
Emergency spending	−0.2402	0.7865	0.1807	−0.4056	−33.3	0.0233	1431

Notes: Two-part overlap-weighted models for zero-heavy spending outcomes, fitted on the primary sample with cluster-robust standard errors at the VARSTR × VARPSU level. Part 1 = logit model for the probability of any positive spending, Pr(Y > 0); Part 2 = generalized linear model with a Gamma family and log link for E[Y | Y > 0] among positive spenders only. Part 1 logit coef = coefficient on sustained GLP-1 use in the Part 1 logit; Part 1 OR = odds ratio computed as exp(coef); Part 1 *p*-value = raw two-sided *p*-value for the Part 1 contrast; Part 2 gamma coef = log-link coefficient on sustained GLP-1 use in the Part 2 Gamma generalized linear model; Part 2% change = exp(coef) − 1, expressed as a percentage, converting the gamma coefficient for ease of interpretation; Part 2 *p*-value = raw two-sided *p*-value for the Part 2 contrast; Positive spenders = number of person-years with Y > 0 in the primary sample. OR = odds ratio; GLP-1 = glucagon-like peptide-1 receptor agonist.

**Table 12:** Cross-specification concordance summary.

Outcome	Specifications	Share Significant at 5%	Share Significant at 10%	Coefficient Range
Acute-care spending	5	100%	100%	−0.5912 to −0.4961
Non-drug medical spending	8	38%	75%	−0.4320 to −0.0937
Total medical spending	5	100%	100%	0.3026 to 0.3195

Notes: Concordance of the GLP-1 contrast across alternative specifications for each primary spending outcome; the concordance summary pools the alternative specifications. Specifications = total number of alternative specifications evaluated for that outcome (any-fill exposure, all-obesity sample, severe-obesity sample, DM-excluded sample, and exploratory molecule-specific specifications); the specification count is outcome-specific because molecule-only exploratory models were available for non-drug spending but not for all other outcomes. The DM-excluded sensitivity sample is included in the concordance count because it is part of the pre-specified robustness panel; nevertheless, because DM-linked GLP-1 use is screened out and treated person-years become very sparse in this configuration (sustained-use *n* = 10), the DM-excluded contributions to the concordance percentages are best read as directional checks rather than as well-powered statistical tests, and the corresponding *p*-values and confidence intervals are correspondingly imprecise. Share significant at 5%/Share significant at 10% = proportion of those alternative specifications in which the GLP-1 contrast attains statistical significance at the 5% and 10% levels, respectively. Coefficient range = minimum and maximum log-link coefficients on sustained GLP-1 use across the alternative specifications, summarizing the directional and magnitude envelope. Companion sign and significant details are reported in Table A2. GLP-1 = glucagon-like peptide-1 receptor agonist; DM = diabetes mellitus. Because these alternative specifications are constructed from the same underlying sample and share most design features, the concordance percentages summarize internal sign stability across related specifications and should not be interpreted as independent replications or as a formal test of replicability.

## Data Availability

The empirical results reported here are derived from public use MEPS data files, as described in Section 3 (https://meps.ahrq.gov/mepsweb/data_stats/download_data_files.jsp, accessed on 15 December 2025). Analytic code is available from the corresponding author upon reasonable request.

## Appendix A

The appendix reports the sequential benchmark estimates and the detailed sensitivity models that underpin the main text’s credibility diagnostics and concordance summaries.

**Table A1:** Sequential benchmark marginal effects for the four primary spending outcomes.

Outcome	M1	M2	M3	M4	M5	M5 *p*-Value
Non-drug medical spending	−3118	−3116	−3143	−2794	−2586	0.0181
Acute-care spending	−2270	−2262	−2271	−2188	−2019	0.003
Total medical spending	5815	5785	5826	6195	6483	0
Out-of-pocket spending	291	297	274	306	325	0.1237

Notes: Marginal dollar contrasts on the sustained GLP-1 indicator under the M1→M2→M3→M4→M5 specification ladder fit as overlap-weighted PPML models on the primary sample, with cluster-robust standard errors at the VARSTR × VARPSU level. M1 is the treatment-only model; M2 adds demographic controls; M3 adds socioeconomic controls; M4 adds clinical controls; and M5 is the full benchmark specification. M5 *p*-value = raw two-sided *p*-value for the M5 GLP-1 contrast. All values are marginal effects in US dollars per person-year. PPML = Poisson pseudo-maximum likelihood; GLP-1 = glucagon-like peptide-1 receptor agonist.

**Table A2:** Detailed sensitivity and exploratory molecule-specific estimates.

Outcome	Specification	β	*p*-Value	Marginal Effect ($/PY)	Treated *n*
Non-drug medical spending	Primary sample—any fill	−0.2489	0.0184	−2584	298
Non-drug medical spending	All obesity—sustained	−0.248	0.0181	−2552	277
Non-drug medical spending	All obesity—any fill	−0.2475	0.0183	−2548	300
Non-drug medical spending	Severe obesity—sustained	−0.2095	0.0868	−2287	183
Non-drug medical spending	Severe obesity—any fill	−0.2097	0.0866	−2291	196
Acute-care spending	Primary sample—any fill	−0.575	0.0035	−1990	298
Acute-care spending	All obesity—sustained	−0.5912	0.0026	−2010	277
Acute-care spending	All obesity—any fill	−0.5803	0.0031	−1981	300
Acute-care spending	Severe obesity—sustained	−0.5093	0.0339	−1953	183
Acute-care spending	Severe obesity—any fill	−0.4961	0.0376	−1908	196
Total medical spending	Primary sample—any fill	0.3132	0	6372	298
Total medical spending	All obesity—sustained	0.3195	0	6446	277
Total medical spending	All obesity—any fill	0.3145	0	6338	300
Total medical spending	Severe obesity—sustained	0.3078	0	6458	183
Total medical spending	Severe obesity—any fill	0.3026	0.0001	6344	196
Non-drug medical spending	Primary sample—semaglutide only	−0.432	0.0785	−3792	62
Non-drug medical spending	Primary sample—liraglutide only	−0.1416	0.3091	−1419	118
Non-drug medical spending	Primary sample—dulaglutide only	−0.0937	0.5309	−955	121

Notes: M5 (fully adjusted) overlap-weighted PPML estimates under each alternative specification fit on the relevant analytic sample, with cluster-robust standard errors at the VARSTR × VARPSU level. Specification = the alternative analytic configuration evaluated in that row (alternative-treatment definitions: “any fill” replaces sustained use with any GLP-1 fill in the person-year; alternative-sample definitions: “all obesity” applies BMI ≥ 30 alone, “severe obesity” applies BMI ≥ 35; molecule-only configurations: “semaglutide only”, “liraglutide-only”, “dulaglutide only” replace the composite GLP-1 indicator with single-molecule indicators). β = log-link coefficient on the relevant GLP-1 indicator under the specification stated in the “Specification” column; *p*-value = raw two-sided *p*-value for the GLP-1 contrast; Marginal effect (USD/PY) = average treated-minus-control predicted spending contrast in US dollars per person-year; Treated *n* = number of treated person-years in the relevant subanalysis. The first fifteen rows summarize alternative sample and treatment-definition specifications for the three main spending outcomes. The final three rows are exploratory molecule-specific models for non-drug medical spending and are included for transparency rather than definitive inference; the corresponding Treated N values are small (semaglutide-only n = 62; liraglutide-only *n* = 118; dulaglutide-only *n* = 121) and these estimates should be interpreted as descriptive rather than inferential, with the molecule-specific *p*-values and confidence intervals understood as underpowered relative to the benchmark contrast in Table 4. PPML = Poisson pseudo-maximum likelihood; GLP-1 = glucagon-like peptide-1 receptor agonist; BMI = body mass index (kg/m^2^).

## Appendix B. Analytical Pipeline: Step-by-Step Code Outline

### Appendix B.1. Purpose and Scope

This appendix complements the high-level pseudocode in Section 3.4 (Algorithm A1) and the workflow diagram in Figure 1. It documents the analytical pipeline as it was implemented with the actual Python packages (versions listed in Appendix B.2), function calls, and parameter choices used to produce the results reported in this study. The content is organized to follow the six steps of Figure 1, so that any reader can trace the prose of Section 3 and the diagram in Figure 1 onto a concrete, reproducible procedure. The full annotated scripts are available from the corresponding author on reasonable request.

**Algorithm A1.** Analytical pipeline (high-level pseudocode)
**Inputs:** MEPS Full-Year Consolidated files (2018–2022); BMI, expenditure, utilization, insurance, and survey-design auxiliary files. **Output:** Estimated GLP-1 contrasts on spending and utilization, with cluster robust inference and sensitivity envelope. **1. Data Preparation** **(**pandas**):** 1.1. Read MEPS files; harmonize DUPERSID and YEAR; replace MEPS sentinel codes (−1, −7, −8, −9, −15) with NaN. 1.2. Construct GLP-1 exposure from RXNAME/RXDRGNAM via molecule and brand keyword matching; aggregate to person-year and define glp1_sustained (≥ 2 fills or ≥ 60 days’ supply) and glp1_any_fill. 1.3. Construct ICD-10 comorbidity flags (E11, I10, E78, I25, I50, N18, J45, F32/F33, F40/F41) and a harmonized DM indicator combining the person-file diagnosis flag with the ICD-10 E11 evidence. 1.4. Construct outcomes: nondrug_medexp = TOTEXP − RXEXP, acute_care_exp = IPTEXP + ERTEXP, total_medexp, oop_exp, plus utilization counts (er_visits, inpatient_discharges, inpatient_nights, office_visits, outpatient_visits). **2. Sample construction:** Restrict to adults (age ≥ 18), to years with observed adult BMI (2019,2021), and to BMI ≥ 30. Build the primary sample (BMI ≥ 30 and ≥ 1 cardiometabolic risk), all-obesity (BMI ≥ 30), severe-obesity (BMI ≥ 35), and DM-excluded (BMI ≥ 30 and diabetes_combined = 0) sensitivity samples. **3. Overlap weighting** **(**scikit-learn**):** Fit a logistic propensity model e(x) = P(GLP1= 1 | X) using sklearn.linear_model.LogisticRegression with the full covariate set. Compute overlap weights as w_0 = 1 − e (x) for treated and w_0 = e (x) for controls. Combine with the MEPS full-year survey weight PERWT_F and normalize so that the resulting final_weight sums to the sample size. **4. Spending estimation** **(**statsmodels**):** For each spending outcome, fit Poisson PML with a log link as sm.GLM(y, X, family=Poisson(link=Log(), var_weights=final_weight). Estimate variance with cluster-robust standard errors at the VARSTR × VARPSU level: model.fit (cov_type = ‘cluster’, cov_kwds = {‘groups’: cluster_id}). Run the M1-M5 specification ladder (treatment only→demographics→SES→clinical→self-rated health). Compute marginal-dollar contrasts by predicting under treated and control regimes. **5. Utilization and two-part estimation:** Fit utilization counts with sm.GLM(…, family=Poisson()) and re-estimate as a robustness check with sm.GLM(…, family=NegativeBinomial()); report incidence rate ratios as exp(β). For zero-heavy spending outcomes, fit a two-part model: Part 1 as a logit on 1 [Y > 0] via sm.GLM (…, family=Binomial()), and Part 2 as a Gamma log-link GLM on the positive subsample via sm.GLM(…, family=Gamma (link=Log()). All parts use the normalized final_weight and the same cov_type=’cluster’ inference. **6. Sensitivity, diagnostics, and reporting:** Re-estimate the benchmark contrasts under (i) glp1_any_fill instead of sustained use, (ii) the all-obesity, severe-obesity, and DM-excluded samples, and (iii) molecule-specific exposures (glp1_semaglutide, glp1_liraglutide, glp1_dulaglutide). Aggregate sign and significance across specifications into a concordance summary, apply Benjamini–Hochberg FDR control separately within two pre-specified test families, the four fully adjusted (M5) primary spending contrasts and the five primary utilization contrasts, using a standard rank-based BH procedure with monotone enforcement and produce diagnostic figures (covariate balance, specification stability, sensitivity envelope, offset decomposition).

### Appendix B.2. Software Environment

All analyses were implemented in Python. The results reported in this paper were verified to reproduce in a Python 3.14 environment with the package versions listed below. The principal libraries are:

- pandas (v3.0.5)/numPy (v2.5.1) → data manipulation, sentinel handling, and outcome construction;
- scikit-learn (v1.9.0) → logistic propensity score model;
- statsmodels (v0.14.6) → generalized linear models (Poisson, Gamma, Binomial, Negative Binomial) and cluster-robust inference;
- matplotlib (v3.10.6) → diagnostic and balance figures.

Multiplicity control (Benjamini–Hochberg FDR) is implemented directly in NumPy following the standard three-step algorithm rather than being imported from an external package; the implementation and a self-validation block are documented in Appendix B.8.3.

The survey-design variance is implemented as a survey-approximate cluster-robust sandwich at the VARSTR × VARPSU level (see Section 3.4.1 and Limitations in Section 6). Exact Taylor-linearized variance estimation, as implemented in Stata svy or the R survey package, is identified as a priority for future replication.

### Appendix B.3. Step 1. Data Extraction and Cleaning

Input: MEPS Full-Year Consolidated files (2018–2022), the BMI/obesity auxiliary file, the medical-expenditure file, the utilization file, the insurance/socioeconomic file, the prescribed-medications file, the medical-conditions file (ICD-10), and the survey-design file (VARSTR, VARPSU, PERWT_F).

Operations:

- Read each file with pandas.read_excel; for the prescribed-medications file (multi-sheet), concatenate sheets row-wise into a single long table.
- Cast DUPERSID to a stripped string and YEAR to integer.
- Replace MEPS sentinel codes ({−1, −2, −3, −7, −8, −9, −15}) with NaN across numeric columns.
- Persist cleaned tables as Parquet under 02_clean_data/ for fast downstream loading.

### Appendix B.4. Step 2. Sample Construction

Input: cleaned MEPS tables joined on (DUPERSID, YEAR).

Operations:

- Build a single master person-year table by left-joining the person file (kisi_verileri) with BMI, expenditure, utilization, insurance, survey, GLP-1 person-year exposure, and ICD-10 person-year comorbidity tables.
- Restrict to adults (age ≥ 18), to years with observed adult BMI
- Define analytic samples:

primary      = adult & yr_ok & has_bmi & (BMI ≥ 30) & cardio risk
all_obesity  = adult & yr_ok & has_bmi & (BMI ≥30)
severe_obese = adult & yr_ok & has_bmi & (BMI ≥35)
DM_excluded  = adult & yr_ok & has_bmi & (BMI ≥30) (diabetes_combined == 0)
where cardio-risk = (diabetes_combined | HIBPDX | CHDDX |
    CHOLDX | icd_htn_i10 | icd_lipid_e78 | icd_chd_i25 | STRKDX)

### Appendix B.5. Step 3. Exposure and Outcome Construction

#### Appendix B.5.1. GLP-1 Exposure

From the prescribed-medications table, flag GLP-1 fills by case-insensitive substring matching against generic and brand keyword lists (semaglutide, liraglutide, dulaglutide, exenatide, tirzepatide, lixisenatide, albiglutide; brand names Ozempic, Wegovy, Rybelsus, Saxenda, Victoza, Trulicity, Byetta, Bydureon, Mounjaro, Zepbound, Adlyxin, Soliqua, Xultophy, Tanzeum) on the RXNAME and RXDRGNAM fields. Aggregate to the person-year by summing fills and positive RXDAYSUP, and define:

glp1_any_fill = 1 if any GLP-1 fill in the person-year
glp1_sustained = 1 if (glp1_rx_count >= 2) OR (glp1_total_daysup >= 60)

Molecule-specific indicators (glp1_semaglutide, glp1_liraglutide, glp1_dulaglutide, glp1_exenatide, glp1_tirzepatide) are constructed analogously and used in the molecule-specific sensitivity analysis.

#### Appendix B.5.2. ICD-10 Comorbidities

In the medical-conditions table, normalize ICD10CDX (uppercase, strip whitespace, drop sentinel codes) and create row-level prefix flags for E11 (T2DM), I10 (HTN), E78 (lipids), I25 (CHD), 150 (HF), N18 (CKD), J45 (asthma), G47 (sleep), F32/F33 (depression), F40/F41 (anxiety), and E66 (obesity). Aggregate to the person-year by taking the maximum of each flag, then construct composite counts (cardiometabolic_count, psych_comorbidity_count, chronic_condition_count).

Harmonize the diabetes indicator across the person-file diagnosis flags (DIABDX, DIABDX_M18) and the ICD-10 E11 evidence:

diabetes_combined = max(diabetes_personfile, icd_t2dm_e11)

#### Appendix B.5.3. Outcomes

nondrug_medexp = TOTEXP − RXEXP
acute_care_exp = IPTEXP + ERTEXP
outpatient_exp = OPTEXP + OBVEXP
total_medexp = TOTEXP
rx_exp = RXEXP
oop_exp = TOTSLF
er_visits = ERTOT
inpatient_discharges = IPDIS
inpatient_nights = IPNGTD
office_visits = OBTOTV
outpatient_visits = OPTOTV
rx_fills_total = RXTOT

### Appendix B.6. Step 4. Overlap Weigthing

Covariate vector × includes age, age^2^, female, year-2021 indicator, race dummies (RACETHX), region dummies, insurance coverage (INSCOV), poverty category (POVCAT), education (HIDEG ≥ 4), marital status, employment, self-rated health (RTHLTH53), self-rated mental health (MNHLTH53), and clinical comorbidity indicators (diabetes_combined, icd_htn_i10, icd_lipid_e78, icd_chd_i25, icd_asthma_j45, icd_depression_f32, icd_anxiety_f41, STRKDX, ARTHDX, CANCERDX).

from sklearn.linear_model import LogisticRegression
lr = LogisticRegression(penalty=’l2’, C=1.0, solver=’lbfgs’, max iter=2000)
lr.fit(X, treatment)         # treatment = glp1_sustained
ps = lr.predict_proba(X)[:, 1]  # propensity score e(x)
ow= np.where(treatment==1, 1-ps, ps)    # overlap weight
final_weight= PERWT_F * ow           # combine with MEPS survey weight
# normalize so that weights sum to N for use as analytical (var_weights) input

w_norm = final_weight * len(final_weight)/final_weight.sum() Balance is assessed via weighted absolute standardized mean differences before and after weighing (Figure 2; Table 3). The convention is that |SMD| ≤ 0.10 indicates acceptable balance.

### Appendix B.7. Step 5. Estimation

#### Appendix B.7.1. Cluster Definition and Survey-Approximate Interference

cluster_id = VARSTR.astype(str) + ‘_’ + VARPSU.astype(str)

All GLM fits use var_weights = w_norm and cov_type=’cluster’ with cow_kwds = {‘groups’: cluster_id}, producing cluster-robust sandwich standard errors at the PSU-within-stratum level. This is the survey-approximate inference framework described in Section 3.4.1.

#### Appendix B.7.2. Spending Models (Poisson PML, Log Link)

import statsmodels.api as sm
from statsmodels.genmod.families import Poisson, Gamma, Binomial, NegativeBinomial
from statsmodels.genmod.families.links import Log
    for outcome in [‘nondrug_medexp’, ‘acute_care_exp’, ‘total_medexp’, ‘oop_exp’]:
      for spec in [‘M1_treatment_only’, ‘M2_demo’, ‘M3_ses’,
         ‘M4_clinical’, ‘M5_full’]:
      X_spec = sm.add_constant(design_matrix(spec))
      model = sm.GLM(y, X_spec,
          family=Poisson(link=Log()),
          var_weights=w_norm)
      fit    = model.fit(cov_type=’cluster’,
             cov_kwds={‘groups’: cluster_id},
              maxiter=200)
      # marginal-dollar contrast
      X1 = X_spec.copy(); X1[:, 1] = 1
      X0 = X_spec.copy(); X0[:, 1] = 0
       marginal_dollar = np.average(fit.predict(X1) – fit.predict(X0),
                weights=w_norm)

The five-model specification ladder (M1 → M5) corresponds to the progressive addition of demographic, socioeconomic, clinical, and self-rated health controls described in Section 3.4.2.

#### Appendix B.7.3. Two-Part Models (Zero-Heavy Spending Outcomes)

# Part 1 – P(Y > 0) via logit
f1 = sm.GLM((y > 0).astype(int), X,
        family=Binomial(),
        var_weights=w_norm)\
     .fit(cov_type=’cluster’, cov_kwds={‘groups’: cluster_id})
# Part 2 – E[Y | Y > 0] via Gamma log-link on the positive subsample
f2 = sm.GLM(y[y > 0], X[y > 0, :],
       family=Gamma(link=Log()),
       var_weights=w_norm[y > 0])\
    .fit(cov_type=’cluster’, cov_kwds={‘groups’ : cluster_id[y >0]})

#### Appendix B.7.4. Utilization Models (Counts)

# Poisson PML benchmark
fp = sm.GLM(y_count, X, family=Poisson(),
          var_weights=w_norm)\
          .fit(cov_type=’cluster’, cov_kwds={‘groups’: cluster_id})
IRR     = np.exp(fp.params[1])
IRR_lo  = np.exp(fp.conf_int()[1, 0])
IRR_hi  = np.exp(fp.conf_int()[1, 1])
# Negative Binomial robustness
fn = sm.GLM(y_count, X, family=Negative Binomial),
          var_weights=w_norm)\
          .fit(cov_type=’cluster’, cov_kwds={‘groups’: cluster_id})

### Appendix B.8. Step 6: Diagnostics, Sensitivity, and Reporting

#### Appendix B.8.1. Sensitivity Envelope

The benchmark M5 specification is re-estimated, for each spending outcome, under the alternative-treatment definition (glp1_any_fill), across the all-obesity, severe-obesity, and DM-excluded sensitivity samples, as well as under the molecule-specific exposures. Each re-estimation uses the same overlap-weighted, cluster-robust pipeline as the benchmark; for the alternative samples, the propensity model is refitted within the relevant subsample.

#### Appendix B.8.2. Concordance Summary

Sign and significance across the alternative specifications are summarized in the sensitivity-concordance table (Table 12) and the sensitivity envelope (Figure 4). Sign reversal in Table 8 is recorded whenever a sensitivity estimate flips sign relative to the benchmark.

#### Appendix B.8.3. Multiplicity Control

FDR control is applied separately within two pre-specified test families: (i) the four fully adjusted (M5) primary spending contrasts (nondrug_medexp, acute_care_exp, total_medexp, oop_exp), reported in Table 4, and (ii) the five primary utilization contrasts (er_visits, inpatient_discharges, inpatient_nights, office_visits, outpatient_visits), reported in Table 11. The Benjamini–Hochberg procedure [29] is implemented directly, using the standard three-step algorithm rather than an external package, and is applied to each family independently:

def benjamini_hochberg (pvals):
    pvals = np.asarray(pvals, dtype=float)
    valid = ~np.isnan(pvals)
    p_valid = pvals[valid]
    m = len(p_valid)

    # 1) sort p-values ascending
    order = np.argsort(p_valid)
    ranks = np.empty_like(order)
    ranks[order] = np.arrange(1, m+1)

    # 2) raw BH adjustment: q_raw(i) = p(i) * (m / rank(i)
     q_raw = p_valid * m / ranks

    # 3) enforce monotonicity from largest to smallest rank,
    #    and cap q-values at 1.0
    sorted_q = q_raw[order]
    sorted_q[-1] = min(sorted_q[-1], 1.0)
    for j in range(len(sorted_q) -2, -1, -1):
        sorted_q[j+1] = min(sorted_q[j + 1], 1.0)
        sorted_q[j]   = min(sorted_q[j], sorted_q[j + 1])
    q_corrected = np.empty_like(sorted_q)
    q_corrected[order] = sorted_q

    q_full        = np.full_like(sorted_q)
    q_full[valid] = q_corrected
    return q_full
# Family 1: M5 primary utilization contrasts (m=4)
q_spending    = benjamini_hochberg(pvals_M5_spending)

# Family 2: primary utilization contrasts (m=5)
q_utilization = benjamini_hochberg(pvals_utilization)

BH-adjusted q-values are reported alongside raw *p*-values in Table 4 (spending) and Table 10 (utilization). The implementation reproduces the q-values published in the main manuscript exactly; the calculation includes a self-validation step that compares the recomputed q-values against the published values for both families.

#### Appendix B.8.4. Diagnostic Features

- Analytical workflow: six-step vertical flow diagram from data extraction to diagnostics, with side panels listing operational details (Figure 1).
- Participant flow: STROBE-compliant flow chart from MEPS source files through inclusion/exclusion steps to the primary and sensitivity samples (Figure A1).
- Covariate balance: weighted love plot of |SMD| before and after overlap weighting (Figure 2).
- Specification stability: coefficient retention from M1 to M5 (Figure 3).
- Sensitivity envelope: sign and magnitude across alternative specifications (Figure 4).
- Offset decomposition: illustrative pharmacy/non-drug/acute-care decomposition (Figure 5) and same-year dollar offset (Figure 6).

### Appendix B.9. Reproducibility Statement

Inputs (the publicly available MEPS Full-Year Consolidated and event files), the cleaning rules summarized above, and the modeling choices described in Section 3 of the main manuscript, together with the package-level operational summary in this appendix, fully specify the analytical pipeline. Annotated Python scripts are available from the corresponding author on reasonable request.
